# Development of Clinically Optimized Sitagliptin and Dapagliflozin Complex Tablets: Pre-Formulation, Formulation, and Human Bioequivalence Studies

**DOI:** 10.3390/pharmaceutics15041246

**Published:** 2023-04-14

**Authors:** So-Jin Kang, Joo-Eun Kim

**Affiliations:** 1 Department of Pharmaceutical Engineering, Catholic University of Daegu, Hayang-Ro 13-13, Gyeongsan 38430, Republic of Korea; sbtmxlsk@naver.com; 2 Department of Biopharmaceutical Chemistry, School of Applied Chemistry, Kookmin University, Seoul 02707, Republic of Korea

**Keywords:** sitagliptin phosphate monohydrate, dapagliflozin propanediol monohydrate, formulation, drug-controlled release, double-layer tablet, human bioequivalence

## Abstract

The purpose of this study is to derive an optimal drug release formulation with human clinical bioequivalence in developing a sitagliptin phosphate monohydrate-dapagliflozin propanediol hydrate fixed-dose combination (FDC) tablet as a treatment for type 2 diabetes mellitus. As a treatment for type 2 diabetes mellitus, the combined prescription of dipeptidyl peptidase-4 (DPP-4) inhibitors and sodium-glucose cotransporter-2 (SGLT-2) inhibitors is common. Therefore, this study simplified the number of individual drugs taken and improved drug compliance by developing FDC tablets containing sitagliptin phosphate monohydrate as a DPP-4 inhibitor and dapagliflozin propanediol hydrate as an SGLT-2 inhibitor. To derive the optimal dosage form, we prepared single-layer tablets, double-layer tablets, and dry-coated tablets and evaluated the drug control release ability, tableting manufacturability, quality, and stability. Single-layer tablets caused problems with stability and drug dissolution patterns. When the dissolution test was performed on the dry-coated tablets, a corning effect occurred, and the core tablet did not completely disintegrate. However, in the quality evaluation of the double-layer tablets, the hardness was 12–14 kilopond, the friability was 0.2%, and the disintegration was within 3 min. In addition, the stability test revealed that the double-layer tablet was stable for 9 months under room temperature storage conditions and 6 months under accelerated storage conditions. In the drug release test, only the FDC double-layer tablet showed the optimal drug release pattern that satisfied each drug release rate. In addition, the FDC double-layer tablet showed a high dissolution rate of over 80% in the form of immediate-release tablets within 30 min in a pH 6.8 dissolution solution. In the human clinical trial, we co-administered a single dose of a sitagliptin phosphate monohydrate-dapagliflozin propanediol hydrate FDC double-layered tablet and the reference drug (Forxiga^®^, Januvia^®^) in healthy adult volunteers. This study showed clinically equivalent results in the stability and pharmacodynamic characteristics between the two groups.

## 1. Introduction

Diabetes mellitus is a phenomenon in which ingested glucose is not absorbed in the body, accumulates in the blood, and is excreted in the urine due to insufficient secretion or poor secretion of insulin for various reasons [1,2]. Diabetes mellitus occurs when the insulin secretion function of the pancreas decreases as the body’s demand for insulin increases [3]. In addition, diabetes mellitus can be associated with congenitally low insulin secretion and is commonly caused by insulin resistance due to genetic/environmental factors. The symptoms of diabetes mellitus include severe thirst, polyuria, and polydipsia [3,4]. Diabetes mellitus is the lack of insulin and can be divided into type 1 diabetes and type 2 diabetes. Type I diabetes mellitus is an insulin-dependent diabetes mellitus that is caused by a deficiency in the secretion of insulin in the pancreas due to the destruction of pancreatic β cells [5,6]. Type I diabetes mellitus requires insulin administration because of the lack of blood sugar control [7]. Type 2 diabetes mellitus is non-insulin-dependent diabetes; in this condition, insulin is secreted by pancreatic β cells, but insulin resistance occurs in the body, causing a decrease in insulin secretion or a breakdown in the insulin response system [4,8]. In particular, type 2 diabetes mellitus has multiple causes, and the symptoms are severe due to β-cell inactivation, insulin resistance, and inflammatory responses due to environmental factors (obesity, dietary habits, lack of exercise) and genetic factors [9]. According to a presentation by the International Diabetes Federation (IDF), the prevalence of diabetes mellitus in adults aged 20 to 79 worldwide was 9.3% as of 2019 [10], and the approximately 285 million patients with diabetes mellitus are estimated to increase to 438 million by 2030 [11]. Approximately 1.5 million people worldwide died in 2019 due to diabetes mellitus. In Korea, the prevalence rate of diabetes mellitus among adults aged 30 years and older was 13.8% in 2018, and the death rate due to diabetes mellitus was 28.9 per 100,000 in 2013 [11]. Diabetes mellitus has been identified as the leading cause of life expectancy decline and death [12].

Sitagliptin phosphate monohydrate ((R)-3-Amino-1-(3-(trifluoromethyl)-5,6-dihydro-[1,2,4]triazolo[4,3-a]pyrazin-7(8H)-yl)-4-(2,4,5-trifluorophenyl)butan-1-one phosphate monohydrate, a treatment for type 2 diabetes mellitus, is the first commercially commercialized oral hypoglycemic agent among dipeptidyl peptidase-4 (DPP-4) inhibitors and is taken as monotherapy or combination therapy at a dose of 100 mg once a day [13]. DPP-4 inhibitors control blood glucose by increasing insulin secretion and suppressing glucagon release by restricting the DPP-4 enzyme that decomposes incretin, a hormone secreted in the gastrointestinal tract when food is ingested [14,15]. DPP-4 inhibitors do not cause weight gain compared with the impact of a rapid decrease in blood glucagon, and because they act dependently on blood glucagon concentrations in the body, they have a low risk of hypoglycemia and are used as an adjuvant to dietary and exercise therapy [15,16]. Sitagliptin phosphate monohydrate, a DPP-4 inhibitor, has a molecular weight of 523.32 g/mol, and has good solubility and permeability as its biopharmaceutics classification system (BCS) is Class 1. In addition, sitagliptin phosphate monohydrate has a time to maximum plasma concentration (Tmax) of 1 to 4 h and has a suitable distribution volume and rapid oral absorption in vivo with a bioavailability of approximately 87% [17]. Dapagliflozin propanediol hydrate ((1S)-1,5-Anhydro-1-C-[4-chloro-3-[(4-ethoxyphenyl)methyl]phenyl]-D-glucitol (S)-propane-1,2-diol (1:1) monohydrate, BMS 512148 propanediol monohydrate, Dapagliflozin (S)-propylene glycol monohydrate, (2S,3R,4R,5S,6R)-2-(3-(4-Ethoxybenzyl)-4-chlorophenyl)-6-hydroxymethyl-tetrahydro-2H-pyran-3,4,5-triol propanediol monohydrate) is a type 2 oral antidiabetic drug that is a sodium-glucose cotransporter-2 (SGLT-2) inhibitor and reduces blood glucose independently of insulin action [18]. SGLT-2 inhibitors reduce glucose reabsorption in the kidneys by inhibiting SGLT-2, which affects glucose reabsorption in the kidneys, and lower blood glucagon by excreting glucose in the urine [19]. In addition, regardless of diabetes mellitus treatment, there is an effect of reducing the risk of hospitalization and death due to chronic heart failure and cardiovascular disease [19,20]. Dapagliflozin propanediol hydrate can be taken at a dose of 10 mg once a day as monotherapy or combination therapy regardless of the meal [21], and the Tmax and bioavailability are 2 h and 78%, respectively [11]. In addition, as its BCS is Class 3, its solubility is good when orally administered [22]. Currently, patients with type 2 diabetes mellitus require combination therapy with other oral drugs before increasing the dose to the maximum dose when the goal of glycemic control is not reached with monotherapy as diabetes mellitus progresses. Therefore, the combination prescription rate of DPP-4 inhibitors and SGLT-2 inhibitors is high for blood glucose control in patients with diabetes mellitus [23]. However, due to the inconvenience of taking two drugs simultaneously, drug compliance is low [24].

Therefore, the purpose of this study is to derive an optimal drug release formulation with human clinical bioequivalence in developing sitagliptin phosphate monohydrate-dapagliflozin propanediol hydrate fixed-dose combination (FDC) tablets, a treatment for type 2 diabetes. We want to conduct a comparative study by manufacturing single-layer tablets, double-layer tablets, and dry-coated tablets that satisfy drug release ability, manufacturability, quality, stability, and dissolution patterns for the two active pharmaceutical ingredients (APIs). In terms of actual tests, each formulation is difficult to evaluate for the clinically desired drug release pattern. The two APIs used in the study have various problems. Practically, sitagliptin phosphate monohydrate has a problem of sticking to the punch due to the high ratio of 100 mg per tablet, and the characteristics of the drug cause agglomeration and friction. In addition, dapagliflozin phosphate monohydrate, despite its low ratio of 10 mg per tablet, is bulky due to its high apparent density and causes blend uniformity problems due to its high cohesion rate. To solve the problems that occur during tableting due to the properties of the API, sitagliptin phosphate monohydrate was prepared by wet granulation, and dapagliflozin propanediol hydrate was prepared by direct compression. In addition, although the FDC tablet has an economical advantage over taking each tablet in combination therapy, problems such as issues with dissolution patterns or stability may occur due to interactions between the APIs. Therefore, it is important to overcome these interaction problems and demonstrate synergy so that excipients with the most stable API can be selected through pre-formulation and compatibility studies. Single-layer tablets, double-layer tablets, and dry-coated tablets were prepared using excipients selected through a compatibility study with the API, and drug dissolution patterns were compared through a dissolution study. Then, the optimal sitagliptin phosphate monohydrate-dapagliflozin propanediol hydrate FDC tablet was selected through the evaluation of the drug release pattern, content, impurity, and stability. The developed sitagliptin phosphate monohydrate-dapagliflozin phosphate hydrate FDC tablet was challenged to enable rapid absorption in the body by promoting rapid disintegration in the gastrointestinal tract when administered orally. In addition, DPP-4 inhibitors and SGLT-2 inhibitors can complement each other with their different action mechanisms, and the risk of hypoglycemia is relatively low, which is expected to reduce side effects and increase the therapeutic effect. Therefore, this study secured safety, stability, and efficacy by deriving the optimal formulation through a comparative study of drug release among single-layer tablets, double-layer tablets, and dry-coated tablets. In addition, the optimal dosage form aimed to simplify the number of drugs taken by patients and to reduce drug costs.

Then, through a human clinical trial, we co-administered a single dose of a sitagliptin phosphate monohydrate-dapagliflozin propanediol hydrate FDC double-layer tablet and the reference drug (Forxiga^®^, Januvia^®^) to healthy adult volunteers, and the safety and pharmacodynamics characteristics of the two groups were evaluated to confirm the bioequivalence.

## 2. Materials and Methods

### 2.1. Materials

Sitagliptin phosphate monohydrate was purchased from Dongbang FTL Ltd. (Hwaseong, Republic of Korea), and dapagliflozin propanediol monohydrate was purchased from Biochem Ltd. (Sejong, Republic of Korea). Dicalcium phosphate anhydrous was provided by Budenheim (Di-cafos A 150, Mainz-Bingen, Rhineland-Palatinate, Germany), and microcrystalline cellulose was supplied by JRS Pharma (Heweten 102, Holzmuhle 1, Rosenberg, Germany). Sodium starch glycolate was supplied by Roquette PTE Ltd. (GLYCOLYS, Roquette Pharma, Lestrem, France), and sodium stearyl fumarate was purchased from Anhui Sunhere Pharma (Huainan, China). Colloidal silicon dioxide was provided by Evonik Industries AG. (Aerosil 200, Essen, Germany), and crospovidone was purchased from BASF (Kollidon CL, Ludwigshafen, Germany). Magnesium stearate was purchased from Faci Asia Pacific Pte Ltd. (Merlimau PI, Jurong Island, Singapore), and silicified microcrystalline cellulose was supplied by JRS Pharma (Prosolv SMCC 90, Holzkohle 1, Rosenberg, Germany). OPADRY II was supplied by Colorcon Asia Pacific Pte Ltd. (Somerset Road, Singapore). Acetonitrile and methanol were purchased in high-performance liquid chromatography (HPLC) grade from Duksan Pharmaceutical Co. Ltd. (Ansan, Republic of Korea). pH 2.0–12.0 buffer was purchased as an extra pure grade from Duksan Pharmaceutical Co. Ltd (Ansan, Republic of Korea). Deionized water was used at 18 MΩ using a distillation device in the laboratory. All other chemicals were of analytical reagent grade and were purchased commercially.

### 2.2. HPLC Analysis

#### 2.2.1. Simultaneous Quantitative Analytical Methods for Assay and Dissolution

The content, content uniformity, and dissolution simultaneous quantitative analyses of sitagliptin phosphate monohydrate-dapagliflozin propanediol hydrate FDC tablets were performed using an HPLC system (Agilent 1260 Infinity II, Agilent Technologies, Santa Clara, CA, USA) equipped with a UV visible detector. The column used was an Agilent C18 column (5.0 μm, 4.6 × 150 mm). The mobile phase and diluted solution of the content and dissolution studies analyses were prepared with 0.5% triethylamine buffer (*v*/*v*, pH 6.8 adjusted with 85% phosphoric acid) and acetonitrile at a ratio of 7:3 (*v*/*v*). The mobile phase was filtered through a 0.45-μm polypropylene membrane filter and degassed via sonication. The wavelength, flow rate, and injection volume were set to 205 nm, 1.5 mL/min, and 20 μL, respectively. In the case of the content and dissolution studies, the mobile phase was constantly flowed for 18 min via an isocratic elution method and analyzed. The peak retention times of sitagliptin phosphate monohydrate and dapagliflozin propanediol hydrate were 2.5 min and 12 min, respectively. We performed method validation of the simultaneous quantitative analytical method for pharmaceutic content and dissolution studies and verified the validity of this method through system suitability, specificity, linearity, accuracy and precision, detection limit and quantitation limit, and solution stability testing.

#### 2.2.2. Simultaneous Quantitative Analytical Method to Assess for Impurities

A simultaneous quantitative analytical method to assess for impurities of sitagliptin phosphate monohydrate-dapagliflozin propanediol hydrate FDC tablets was performed using an HPLC system (Agilent 1260 Infinity II, Agilent Technologies, USA) equipped with a UV visible detector. The column used was the Gemini C18 column (5.0 μm, 250 mm). Mobile phase A of the impurity study was prepared with KH2PO4 buffer (*v*/*v*, solution adjusted to pH 6.0 with 1 M KOH): acetonitrile: methanol = 85: 10: 5 (*v*/*v*/*v*), and mobile phase B was prepared with 90% acetonitrile. The mobile phase was filtered through a 0.4 μm polypropylene membrane filter and degassed via sonication. The diluted solution was prepared with KH2PO4 buffer (*v*/*v*, solution adjusted to pH 6.0 with 1 M KOH) and acetonitrile at a ratio of 7:3 (*v*/*v*). The wavelength, flow rate, and injection volume were set to 220 nm, 1.0 mL/min, and 20 μL, respectively. The column and sample thermostats were set to 35 °C and 5 °C, respectively. In the case of the impurity study, the analysis was performed by changing the composition of the mobile phase for 80 min using a gradient elution method for 80 min. The peak retention times of sitagliptin phosphate monohydrate and dapagliflozin propanediol hydrate were 16 min and 37 min, respectively. We performed method validation of the simultaneous quantitative analytical method for the pharmaceutic impurity study and verified the validity of this method through system suitability, specificity, linearity, accuracy and precision, detection limit and quantitation limit, and solution stability testing.

### 2.3. Solubility Study for the Two APIs

**Figure 1:**
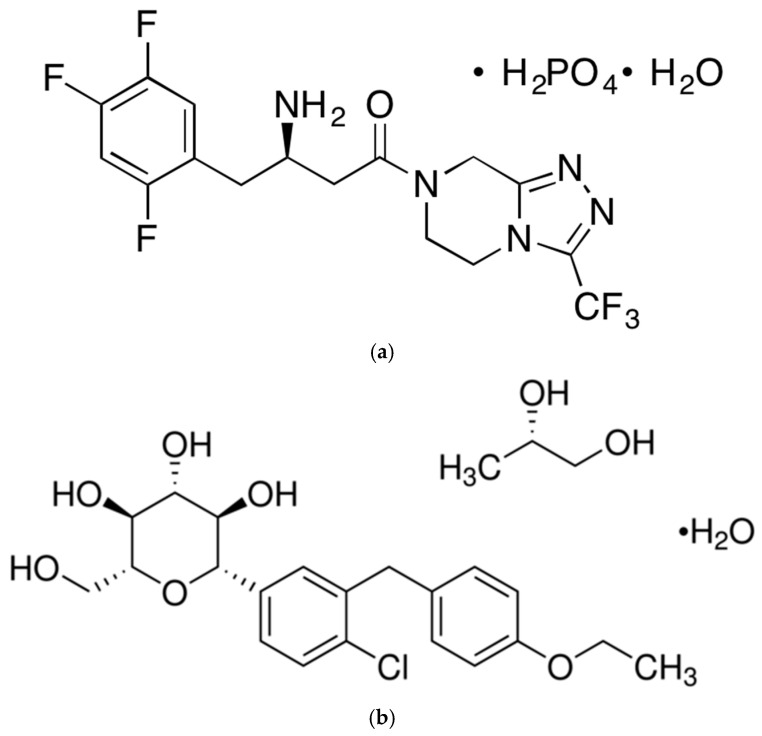
Chemical structure of sitagliptin phosphate monohydrate (**a**) and dapagliflozin propanediol hydrate (**b**).

As shown in Figure 1, the solubility of sitagliptin phosphate monohydrate and dapagliflozin propanediol hydrate as APIs was evaluated sequentially using the apparent solubility test method and the equilibrium solubility test method according to USP <1236> solubility measurements [25]. The solubility of the two APIs was evaluated in pH 1.2 to 12.0 buffer solutions, deionized water, ethanol, methanol, and acetonitrile.

Ten milligrams of each sitagliptin phosphate monohydrate and dapagliflozin propanediol hydrate were weighed and put in each beaker. Then, 200 μL of each solvent was added and stirred at 400 rpm. The temperature of the sample was maintained at 20 ± 2 °C. After shaking for 30 s every 5 min, 200 μL of each solvent was added until no particles of the API were visible. The apparent solubility of the two main components was estimated through the point at which all of the API dissolved and became clear.

The equilibrium solubility test was used to calculate the input amount through the apparent solubility test result, and an excess of the API was placed in each beaker and stirred at 400 rpm. At 0, 12, and 24 h immediately after the overdose, 2 mL samples were taken and filtered through a 0.45-μm regenerated cellulose syringe filter. The supernatant of the filtered solution was taken and diluted with a content analysis diluted solution. The solubility of sitagliptin phosphate monohydrate and dapagliflozin propanediol hydrate was calculated from the peak areas obtained from the sample and standard solutions using an HPLC system (Agilent 1260 Infinity II, Agilent Technologies, USA) according to the simultaneous quantitative analytical method. A calibration test was performed, and the sample was diluted with a diluted solution and prepared at a concentration of 0.05–1 mg/mL. The standard deviation (SD) of accuracy and precision was less than 2%, and the correlation coefficient of the calibration curve was 1, which was linear.

### 2.4. Stability Study According to pH Buffer for the Two APIs

Sitagliptin phosphate monohydrate and dapagliflozin propanediol hydrate as APIs were prepared at concentrations of 4 μg/mL and 0.4 μg/mL, respectively, using pH buffers (pH 1.2 to 12.0). Each shaded sample was stored for 4 weeks under a stability chamber under room temperature storage conditions (25 ± 2 °C/60 ± 5% relative humidity) and accelerated storage conditions (40 ± 2 °C/75 ± 5% relative humidity). The amount of total impurity in sitagliptin phosphate monohydrate and dapagliflozin propanediol hydrate was analyzed using an HPLC system (Agilent 1260 Infinity II, Agilent Technologies, USA) according to impurity simultaneous quantitative analysis. We performed a calibration test, and the sample was diluted with a diluted solution and prepared at a concentration of 0.05–1 mg/mL. The standard deviation (SD) of accuracy and precision was less than 2%, and the correlation coefficient of the calibration curve was linear at 1.0000.

### 2.5. Compatibility Study

The compatibility study was performed using appearance and impurity tests to confirm the interaction between each API and the excipient. Each API was mixed with mannitol, microcrystalline cellulose, lactose, silicified microcrystalline cellulose, calcium hydrogen phosphate anhydrous, pregelatinized starch, sodium starch glycolate, crospovidone, sodium croscarmellose, magnesium stearate, colloidal silicon dioxide, sodium stearyl fumarate, hydroxypropyl cellulose, calcium hydroxide, citric acid, and OPADRY II pink in a 1:1 (*w*/*w*) ratio and put into each clear glass vial. Sitagliptin phosphate monohydrate and dapagliflozin propanediol hydrate were mixed at 100:10 (*w*/*w*), 50:10 (*w*/*w*), and 10:10 (*w*/*w*) ratios and put into a clear glass vial. Sitagliptin phosphate monohydrate-dapagliflozin propanediol hydrate 100:10 (*w*/*w*) refers to the amount of API contained per tablet. Each shaded sample was stored for 4 weeks under the stability chamber of temperature storage conditions (25 ± 2 °C/60 ± 5% relative humidity) and accelerated storage conditions (40 ± 2 °C/75 ± 5% relative humidity). Samples were analyzed for appearance and impurity at 0, 2, and 4 weeks. The amount of total impurity in sitagliptin phosphate monohydrate and dapagliflozin propanediol hydrate was analyzed using an HPLC system (Agilent 1260 Infinity II, Agilent Technologies, USA) according to impurity simultaneous quantitative analysis.

### 2.6. Formulation Studies on Sitagliptin-Dapagliflozin FDC Tablets

Through the compatibility test, the excipient was selected as stable when mixed with the API. Sitagliptin phosphate monohydrate was prepared by wet granulation compression to improve the flowability and content uniformity, and dapagliflozin propanediol hydrate was prepared by direct compression due to problems in flowability, content uniformity, and stability of the API. The composition of the tablet is shown in Table 1.

#### 2.6.1. Manufacture of Sitagliptin Granules

**Figure 2:**
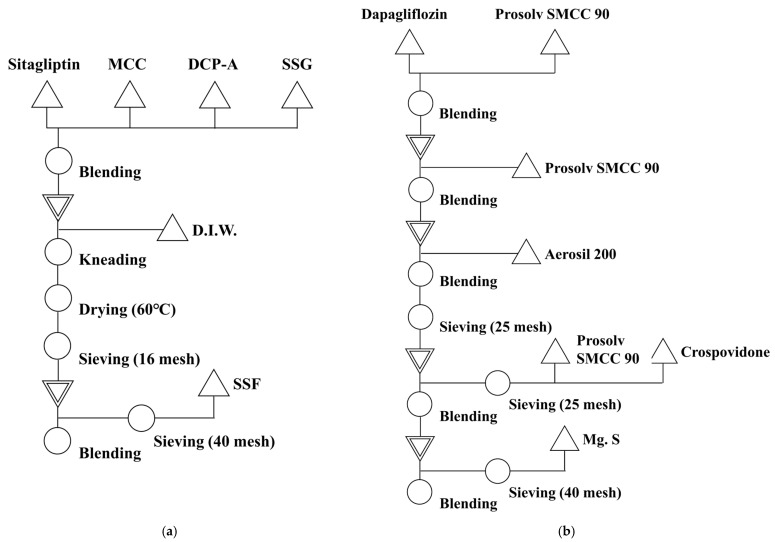
Manufacturing process flow chart of sitagliptin phosphate monohydrate granules (**a**) and dapagliflozin propanediol hydrate mixture powder (**b**).

Sitagliptin phosphate monohydrate was prepared into wet granules using a high-shear mixer (YC-SMG-3, YENCHEN MACHINERY Co., Ltd., Taoyuan City, Taiwan); this is shown in Figure 2. Sitagliptin phosphate monohydrate was mixed with microcrystalline cellulose, calcium hydrogen phosphate anhydrous, and hydroxypropyl cellulose at high speed, and the speeds of the chopper and impeller were set at 100 and 150 rpm, respectively. The prepared wet granules were dried in a drying oven (ED-CO72, Eden meter, Seoul, Republic of Korea) at 60 °C until the moisture content was less than 2.0%. The dried granules were sized through a 16-mesh sieve to improve flowability and reduce the separation between particles. Finally, sodium stearyl fumarate was sieved through a 40-mesh sieve and mixed with the sieved granules.

#### 2.6.2. Manufacture of the Dapagliflozin Mixture

To improve flowability and content uniformity, dapagliflozin propanediol hydrate was mixed one, two, and three times with silicified microcrystalline cellulose for a comparative evaluation (Figure 2). Therefore, we marked the serial dilution of dapagliflozin by dividing it into one serial dilution, two serial dilutions, and three serial dilutions.

A preliminary first mixture was prepared by mixing dapagliflozin propanediol hydrate and silicified microcrystalline cellulose at a ratio of 1:1 (*w*/*w*). The first mixture was prepared by mixing the preliminary first mixture and colloidal silicon dioxide and sieving through a 25-mesh sieve. Subsequently, the mixture was prepared by combing the first mixture with silicified microcrystalline cellulose, crospovidone, and hydroxypropyl cellulose and then sieving through a 25-mesh sieve. Finally, the M1 final mixture of one serial dilution was prepared by sieving magnesium stearate through a 40-mesh sieve and mixing it with the mixture.

A first mixture was prepared by mixing dapagliflozin propanediol hydrate and silicified microcrystalline cellulose in a ratio of 1:1 (*w*/*w*). Then, a preliminary second mixture was prepared by mixing the first mixture and silicified microcrystalline cellulose in a ratio of 1:1 (*w*/*w*). The second mixture was prepared by mixing the preliminary second mixture and colloidal silicon dioxide and sieving through a 25-mesh sieve. Subsequently, the mixture was prepared by combining the second mixture with silicified microcrystalline cellulose, crospovidone, and hydroxypropyl cellulose and then sieving through a 25-mesh sieve. Finally, the M2 final mixture of two serial dilutions was prepared by sieving magnesium stearate through a 40-mesh sieve and combining it with the mixture.

The first mixture was prepared by combining dapagliflozin propanediol hydrate and silicified microcrystalline cellulose at a ratio of 1:1 (*w*/*w*). Then, a preliminary second mixture was prepared by combining the first mixture and silicified microcrystalline cellulose at a ratio of 1:1 (*w*/*w*). The second mixture was prepared by combining the preliminary second mixture and colloidal silicon dioxide and sieving through a 25-mesh sieve. Then, a preliminary third mixture was prepared by combining the second mixture and silicified microcrystalline cellulose in a ratio of 1:1 (*w*/*w*). The third mixture was prepared by combining the preliminary third mixture and colloidal silicon dioxide and sieving through a 25-mesh sieve. Subsequently, the mixture was prepared by combining the third mixture with silicified microcrystalline cellulose, crospovidone, and hydroxypropyl cellulose and then sieving through a 25-mesh sieve. Finally, the M3 final mixture of three serial dilutions was prepared by sieving magnesium stearate through a 40-mesh sieve and combining it with the mixture.

#### 2.6.3. Flowability Test

Sitagliptin phosphate monohydrate granules and dapagliflozin propanediol hydrate mixtures were evaluated using CI and the HR values using bulk density (BD) and tapped density (TD) to determine the amount of powder that could fit into a space such as a high-shear mixer or the hopper of the tablet press [25]. It filled the granulate and mixture to the marked line of a 100-mL mass cylinder to measure the BD. The apparent density was calculated from the weight and volume of the granules. Then, the cylinder filled with granules was tapped to a certain height, and the volume, when the volume did not change, was measured. The TD was calculated using the same weight of the BD and the changed volume. The flowability and flow characteristics of granules and mixtures were measured through the BD and TD values. CI and HR values were evaluated for flowability according to the formula below.

(1) $$\mathrm{Bulk}\;\mathrm{density}\;\left(\mathrm{BD}\right)=\frac{\mathrm{Mass}\;\mathrm{taken}}{100\;\mathrm{mL}}$$

(2) $$\mathrm{Tapped}\;\mathrm{density}\;\left(\mathrm{TD}\right)=\frac{\mathrm{Mass}\;\mathrm{taken}}{\mathrm{Tapped}\;\mathrm{volume}}$$

The CI value is an indicator of the flowability of granules and was calculated with the formula below.

(3) $${\mathrm{Carr}}^{'}s\;\mathrm{index}\;\left(\mathrm{CI}\right)=\frac{\mathrm{TD}-\mathrm{BD}}{\mathrm{TD}}\times 100$$

The HR values were calculated using TD and BD values.

(4) $$\mathrm{Hausner}\;\mathrm{ratio}\;\left(\mathrm{HR}\right)=\frac{\mathrm{TD}}{\mathrm{BD}}$$

#### 2.6.4. Manufacture of Sitagliptin-Dapagliflozin FDC Single-Layer Tablets

**Figure 3:**
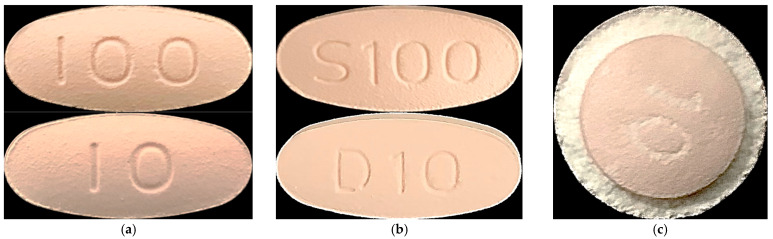
Morphology of three dosage forms. (**a**) Single-layer tablets, (**b**) Double-layer tablets, (**c**) Dry-coated tablets.

Sitagliptin phosphate monohydrate-dapagliflozin propanediol hydrate FDC single-layer tablets were prepared by adsorbing the dapagliflozin propanediol hydrate mixture to sitagliptin phosphate monohydrate granules considering the interaction between the APIs (Figure 3). F1 to F3 FDC single-layer tablets were prepared using a 15.6 × 7.8 mm oval punch and a rotary compression machine (PR-LM, PTK, Gimpo, Republic of Korea). The hardness of the FDC single-layer tablets was set from 12 kp to 14 kp.

#### 2.6.5. Manufacture of Sitagliptin-Dapagliflozin FDC Double-Layer Tablets

Sitagliptin phosphate monohydrate-dapagliflozin propanediol hydrate FDC double-layer tablets are immediate-release tablets with sitagliptin phosphate monohydrate as the lower layer and dapagliflozin propanediol hydrate as the upper layer (Figure 3). FDC double-layer tablets have content non-uniformity and layer separation. To solve this problem, the existence and nonexistence of hydroxypropyl cellulose and the ratio of excipients were adjusted and confirmed. The F4 FDC tablet contained hydroxypropyl cellulose, and the F5 and F6 FDC tablets did not contain hydroxypropyl cellulose. In addition, the dissolution patterns of the F5 and F6 FDC tablets were confirmed by adjusting the weights of the upper and lower layers. FDC double-layer tablets were prepared using a 15.6 × 7.8 mm oval punch and a rotary compression machine (PR-LD, PTK, Gimpo, Republic of Korea). The hardness of the upper and lower layers of the F4 to F6 FDC double-layer tablets affects the drug release and may damage the tablet during distribution. Therefore, the compression pressure of the FDC double-layer tablets was set to 1.0 KN or less and 12 KN or more for the pre-pressure and main pressure, respectively.

#### 2.6.6. Manufacture of Sitagliptin-Dapagliflozin FDC Dry-Coated Tablets

Sitagliptin phosphate monohydrate-dapagliflozin propanediol hydrate FDC dry-coated tablets are immediate-release tablets with sitagliptin phosphate monohydrate as the outer layer and dapagliflozin propanediol hydrate as the inner core (Figure 3). The weight ratio of the outer layer and the inner core should be 1:1.5 to 1:2.5 (*w*/*w*). The inner core tablets were compressed using a 7.0 mm circular punch and a rotary compression machine (PR-LM, PTK, Gimpo, Republic of Korea). The hardness of the inner core tablets was set to 3–4 kp and was seal coated using a PVP coating agent. F7 to F9 FDC dry-coated tablets were prepared using a 10.5 mm circular punch and a rotary compression machine (PR-LT, PTK, Gimpo, Republic of Korea). The hardness of the FDC dry-coated tablets was set from 12 kp to 14 kp.

### 2.7. Risk Assessment for Compressibility Study

In the tableting process of sitagliptin phosphate monohydrate-dapagliflozin propanediol hydrate FDC double-layer tablets, critical process parameters (CPPs) that affect critical material attributes (CQAs) were selected through risk assessment (RA) [26,27,28,29,30]. First, after confirming the risk, factors affecting CQAs in the tableting process were identified through RA tools such as preliminary hazard analysis (PHA) and failure mode effect analysis (FMEA) [31,32]. PHA classified and evaluated risk by color according to the degree of risk. Red represents an unacceptable risk, yellow is an acceptable risk, and green is a broadly acceptable risk [33]. FMEA is graded by probability (P), severity (S), and detectability (D) [34]. If the score of risk priority number (RPN) = P × S × D exceeds 30 [35], the critical process parameter (CPP) among many process parameters (PPs) is selected as the X value [36]. The X value was selected as the risk of taking alternatives such as the design of experiments (DoE). The DoE was evaluated using Minitab software (Version 19; Minitab^®^, Pennsylvania, USA) [37]. The DoE was evaluated through 2-level full factorial design on which parameters had the most influence on the response value and what factor setting was needed to optimize the response value. The X value (input) was selected as compression pressure, turret speed, and feeder speed, and the level of the X value was designed in the area of a compression pressure of 6 to 18 kN, a turret speed of 5 to 15 rpm, and a feeder speed of 10 to 45 rpm (Table 2). As for the Y value (output), it was determined that the weight of tablet, hardness, average of content, deviation of content, and content uniformity acceptance value were the main parameters that could be optimized during the tableting process. According to the 2-level full factorial design, 8 (23) experiments were randomly conducted. Through full factorial design, it was confirmed which factors in the Pareto chart had a significant effect on the response, and if the baseline for the significant level (α = 0.05) was exceeded; this indicted statistical significance. In addition, *p*-values for effects and coefficients in the coded coefficient table were used to decide statistically significant terms at the significance level (α = 0.05). Then, main effect plots and interaction plots were confirmed through factor plots. In the main effect plot, it was judged that the larger the slope of the straight line is, the greater the effect on the Y value, and the interaction plot indicates that there is no interaction effect when the two straight lines are close to parallel. Then, an appropriate design space (DS) was set using contour plots and overlaid contour plots. Through this, when an issue occurs, it is possible to find a solution to the problem and manage the risk to produce a uniform quality.

### 2.8. Quality Evaluation of Three Dosage Forms for Granules and Tablets

The dapagliflozin M1 to M3 mixtures and the FDC F1 to F9 tablets were evaluated considering the hardness, disintegration, and friability.

#### 2.8.1. Hardness Test

The hardness of the tablet has a considerable influence on the release pattern, disintegration, and friability of the drug and can predict whether the tablet will be damaged during transportation [38]. Hardness tests were conducted to determine the crack and production processes of the F1 to F9 tablets. The hardness test was measured using a hardness tester (YD-II, Goldenwall, USA). The tablets were placed on the edges of the fixed and movable parts of the instrument and measured with 10 samples each. Hardness levels are expressed in kilopond (kp). In this experiment, the hardness of the core tablet was set to 3 to 4 kp based on a weight of 200 mg or less, and the hardness of the single-layered tablet, double-layer tablet, and dry-coated tablet were determined to be 12 to 14 kp based on a weight of 500 to 550 mg.

#### 2.8.2. Disintegration Test

The degree of disintegration of the F1 to F9 tablets was measured under the prescribed conditions and time of the test solution. The disintegration test was measured using a disintegration tester (BJ-Ⅰ, Nanbei Instrument Ltd., Zhengzhou, China) according to USP <701> Disintegration. For the disintegration test, six samples were placed in beakers with one tablet each. Next, after filling the beaker with water, the measurement was started at 37 ± 0.5 °C, and the disintegration time was confirmed.

#### 2.8.3. Friability Test

Friability testing of the tablets was performed to predict the tendency to break that may occur during the distribution process. The friability test was conducted to measure the physical strength of the F1 to F9 tablets. Friability was measured using a friability tester (CS-4, Minhua Pharmaceutical Machinery Co., Ltd., Shanghai, China). The friability test was measured with 10 samples, and the tablets were placed in a friability tester and rotated a total of 100 times for 4 min at 25 rpm. Friability was calculated by measuring the weight of tablets before and after testing. According to USP <1216> Tablet friability, it was judged to be suitable if the mass reduction was 1% or less [39].

(5) $$\mathrm{Friability}\;\left(\%\right)=\frac{W_{1}-W_{2}}{W_{2}}\times 100\left(\%\right)$$

W_1_ and W_2_ are the tablet weights before and after testing, respectively.

### 2.9. In Vitro Drug Release Pattern Study

The in vitro comparative dissolution profiles of sitagliptin phosphate monohydrate-dapagliflozin propanediol hydrate FDC tablets and the reference drug were evaluated using a drug dissolution tester (708-DS, Agilent Technologies, USA). The reference drugs for sitagliptin phosphate monohydrate and dapagliflozin propanediol hydrate were Januvia (100 mg tab) and Forxiga (10 mg tab), respectively. The in vitro dissolution test was performed on Apparatus 2 (Paddle Apparatus) of USP <711> Dissolution, and the paddle speed was set to 75 rpm [40]. In addition, pH 6.8 buffer was used as the dissolution solution. The volume and temperature of the dissolution solution were maintained at 900 mL and 37.0 ± 0.5 °C, respectively. The test was performed with 6 tablets each, and 3 mL of the sample solution was collected at regular intervals of 5, 10, 15, 30, 45, and 60 min. The retrieved sample solution was filtered through a 0.45-μm regenerated cellulose syringe filter. The concentrations of sitagliptin phosphate monohydrate and dapagliflozin propanediol hydrate in the filtered sample solution were analyzed using an HPLC system (Agilent 1260 Infinity II, Agilent Technologies, USA) equipped with a UV visible detector according to simultaneous quantitative analysis. Then, the in vitro dissolution profiles of the reference drug and the test drug were compared using the similarity factor (f_2_) recommended for dissolution profile comparison by the Food and Drug Administration (FDA) [41,42]. The similarity factor was calculated using the average dissolution rate (%) between the dissolution curves [43].

(6) $$\mathrm{Similarity}\;\mathrm{factor}\;\left(f_{2}\right)=50\times \log{\left[1+\frac{1}{n}\overset{n}{\underset{i=1}{\sum }}{\left(R_{i}-T_{i}\right)}^{2}\right]}^{-0.5}\times 100$$

n is the number of time points, and $T_{i}$ and $R_{i}$ represent the average dissolution rate of the test drug and reference drug, respectively, at each time point. If the f_2_ value is less than 50%, the dissolution profile is considered to be significantly different. If f_2_ is 50% to 100%, the dissolution pattern between the test drug and the reference drug is considered to be similar.

### 2.10. Stability Study

For the selection of stable sitagliptin phosphate monohydrate-dapagliflozin propanediol hydrate FDC tablets, stability was compared and evaluated by conducting tests on the appearance, assay, and impurity of the F1 to F9 tablets and the reference drug. The test drug and reference drug were subjected to stability tests according to the ICH guideline Q1A. The tablets were stored in a stability chamber at room temperature storage conditions (25 ± 2 °C/60 ± 5% relative humidity) and accelerated storage conditions (40 ± 2 °C/75 ± 5% relative humidity) for 3 months. In addition, the F6 FDC double-layer tablet was studied for 9 months under room temperature storage conditions (25 ± 2 °C/60 ± 5% relative humidity) and 6 months under accelerated storage conditions (40 ± 2 °C/75 ± 5% relative humidity). The assay test, content uniformity test, impurity test, and dissolution test were conducted using 10, 10, 10, and 6 samples, respectively. The stability test was performed using an HPLC system (Agilent 1260 Infinity II, Agilent Technologies, USA) equipped with a UV visible detector according to simultaneous quantitative analysis for assay and impurity.

### 2.11. Human Bioequivalence Study

**Figure 4:**
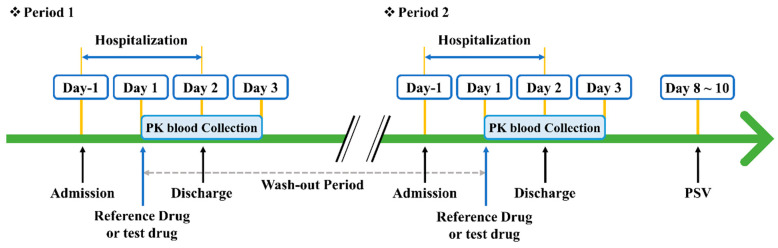
Schematic of the simple two-way crossover design trial.

A phase 1 clinical trial was conducted in the form of a randomized, open-label, single-dose, and two-way crossover study to compare and evaluate the stability and pharmacokinetics of sitagliptin phosphate monohydrate-dapagliflozin propanediol hydrate FDC double-layer tablets and the reference drugs (Forxiga 10 mg tab and Januvia 100 mg tabs) administered to healthy adult volunteers (Figure 4). A total of 40 healthy adult volunteers participated in this study and received one or more doses of the investigational new drug. Only volunteers who voluntarily consented after hearing the explanation about the clinical trial were screened within 4 weeks (Day 28–Day 1) from the first administration date (Day 1 of the first phase) and underwent a health medical examination, vital sign evaluation, physical examination, 12-lead electrocardiography, and clinical laboratory tests. Through this, we selected subjects judged suitable for the clinical trial. Subjects were administered one sitagliptin-dapagliflozin FDC double-layer tablet alone or co-administered one tablet each on the date of administration (Day 1) of the investigational new drug for each period according to the randomly assigned order group. The drug was given as a single dose orally with 150 mL of water. The drug washout period between period 1 and period 2 was 7 days. The blood sampling times of the test drug and Januvia (100 mg tab) were before administration (0 h) and 0.167, 0.33, 0.5, 0.75, 1, 1.5, 2, 3, 4, 5, 6, 8, 12, and 24 h after administration, and the blood sampling times for Forxiga (10 mg tab) were before administration (0 h) and 0.167, 0.33, 0.5, 0.75, 1, 1.5, 2, 3, 4, 6, 8, 12, and 24 h after administration. Blood was collected into an EDTA-K2 tube, and physiological saline was injected to prevent blood coagulation from remaining in the catheter. The analysis of sitagliptin phosphate monohydrate and dapagliflozin propanediol hydrate in the blood was measured using liquid chromatography-tandem mass spectrometry (LC-MS/MS). Since the log-transformed AUC0-t and Cmax were used for pharmacokinetic evaluation variables, the least square mean difference and 90% confidence interval were exponentiated and converted into a geometric mean ratio and its 90% confidence interval. When the transformed 90% confidence interval was within the range of log 0.8 to log 1.25, it was judged that there was no difference in pharmacokinetic characteristics between the two groups.

## 3. Results

### 3.1. Solubility Study for the Two APIs

**Figure 5:**
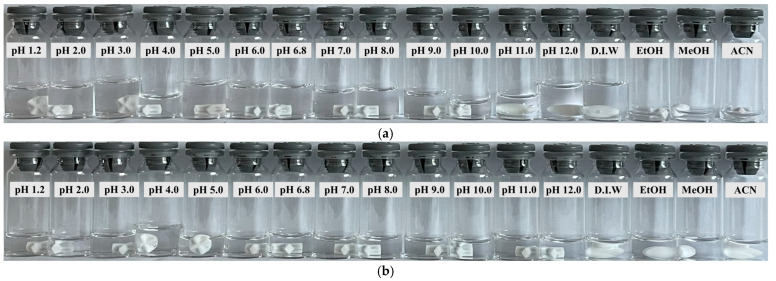
Changes in the appearance of (**a**) sitagliptin phosphate monohydrate and (**b**) dapagliflozin propanediol hydrate in various solvents (apparent solubility test method).

The solubility of sitagliptin phosphate monohydrate and dapagliflozin propanediol hydrate in various solvents is shown in Figure 5 and Table 3 and Table 4.

As a result of the apparent solubility test method, the solubility of sitagliptin phosphate monohydrate in ethanol, methanol, and acetonitrile was evaluated as ‘practically insoluble’ according to the USP <1236> Solubility. However, in other solvents, Sitagliptin phosphate monohydrate was evaluated as ‘soluble’ in USP, and it was confirmed that it had relatively high solubility compared with buffer solutions in other pH ranges including in strong acids of pH 1.2. Overall, sitagliptin phosphate monohydrate was confirmed as a soluble material with high solubility in the aqueous phase. As a result of the equilibrium solubility test method, the solubility in water and pH 1.2–12.0 confirmed high solubility values based on USP standards. However, it showed low solubility in ethanol, methanol, and acetonitrile.

As a result of the apparent solubility test method, the solubility of dapagliflozin propanediol hydrate in methanol was evaluated as ‘freely soluble’ according to the USP <1236> Solubility, and the solubility of ethanol and acetonitrile was evaluated as ‘soluble’. However, it was confirmed that dapagliflozin propanediol hydrate was ‘very slightly soluble’ in other solvents except for the abovementioned solvent. Overall, dapagliflozin propanediol hydrate was identified as a poorly water-soluble drug with low solubility in an aqueous solution. Equilibrium solubility test results showed that it was ‘very slightly soluble’ in water and pH solution. However, as an API that dissolves more than 0.33 mg per 1 mL in 900 mL of dissolution medium, it is likely to be a favorable condition for dissolution testing and immediate release.

As a result, it was confirmed that there was no problem in preparing immediate-release tablets for the two APIs, and they had very appropriate solubility to operate within 500 to 1500 mL of gastric fluid. Therefore, at the present level of solubility, sitagliptin and dapagliflozin are well suited for preparing immediate-release tablets.

### 3.2. Stability Study According to pH Buffer for the Two APIs

Under room temperature storage conditions (25 ± 2 °C/60 ± 5% relative humidity) and accelerated storage conditions (40 ± 2 °C/75 ± 5% relative humidity), sitagliptin phosphate monohydrate and dapagliflozin propanediol hydrate as AIPs were evaluated for impurity tests in various pH solutions for 1 week, and the results are listed in Table 5. Sitagliptin phosphate monohydrate dissolved in pH 3.0 buffer did not change significantly for 3 days under room temperature storage conditions, but rapid decomposition occurred under accelerated storage conditions. Under room temperature storage conditions (25 ± 2 °C/60 ± 5% relative humidity) and accelerated storage conditions (40 ± 2 °C/75 ± 5% relative humidity), the total impurity amount for each pH solution of dapagliflozin propanediol hydrate showed a tendency to increase rapidly under the influence of temperature for a short period of time. However, sitagliptin phosphate monohydrate and dapagliflozin propanediol hydrate showed lower levels of total impurity amount than other solutions in pH 4.0 and pH 6.8 buffers, respectively. Through this, it was confirmed that the two APIs were unstable even for a short time at low or high pH values and that the decomposition rate decreased and the stability improved at neutral pH. This was found to be stable as an intrinsic pH range of sitagliptin phosphate monohydrate and dapagliflozin propanediol hydrate. Therefore, to minimize the decomposition of the drug, the pH of the analysis solution for the impurity and assay tests was set to 6.0 and 6.8, respectively. These results confirmed that dapagliflozin propanediol hydrate is a heat-labile drug in pH buffer, which is the result of the pre-formulation study.

### 3.3. Compatibility Study

**Figure 6:**
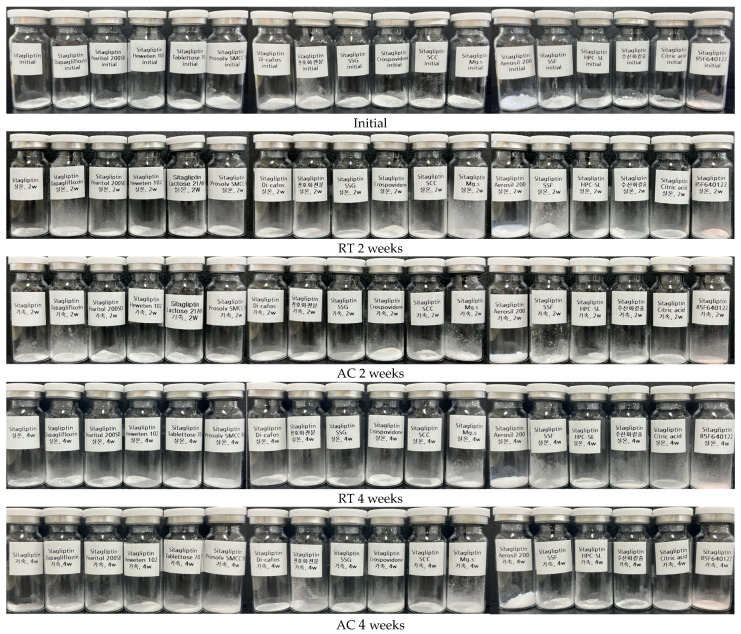
Appearance of compatibility test between sitagliptin phosphate monohydrate and various excipients; RT, room temperature storage condition; AC, accelerated storage condition.

**Figure 7:**
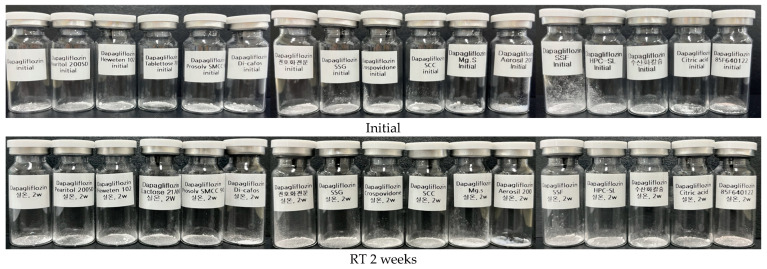

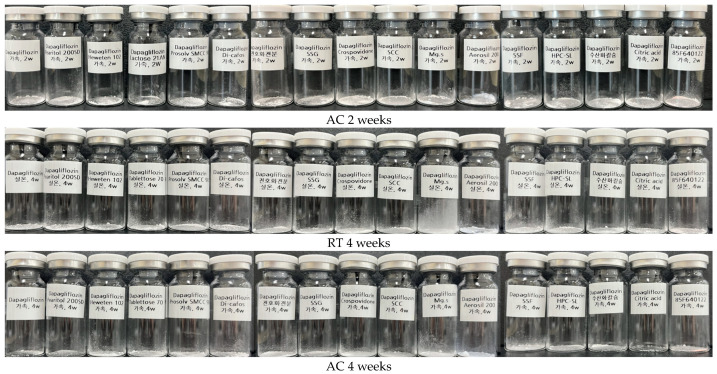
Appearance of compatibility test between dapagliflozin propanediol hydrate and various excipients; RT, room temperature storage condition; AC, accelerated storage condition.

Compatibility studies between the APIs and excipients are important in the preformulation of all formulation development. The API-excipient compatibility test can confirm the effect on the bioavailability and stability of the drug due to the physical/chemical interaction between the API and the excipient, helping to avoid sudden problems. Sitagliptin phosphate monohydrate and dapagliflozin propanediol hydrate were mixed 1:1 (*w*/*w*) with each excipient and stored in room temperature conditions (25 ± 2 °C/60 ± 5% relative humidity) and accelerated storage conditions (40 ± 2 °C/75 ± 5% relative humidity) for 4 weeks. The stored samples were tested for appearance and impurity, and the findings are shown in Table 6 and Table 7. In the compatibility study, there was no change in the appearance of the API-excipient mixture, but the appearance alone does not determine stability (Figure 6 and Figure 7). In the 1:1 (*w*/*w*) mixture of sitagliptin phosphate monohydrate and dapagliflozin propanediol hydrate, related substances rapidly increased to 1.32% and 1.34%, respectively, as a result of storage at room temperature (25 ± 2 °C/60 ± 5% relative humidity) and accelerated storage conditions (40 ± 2 °C/75 ± 5% relative humidity) for 4 weeks. In addition, the 10:1 (*w*/*w*) mixture of sitagliptin phosphate monohydrate and dapagliflozin propanediol hydrate showed a tendency to gradually increase the decomposition, and contact between the APIs in tablet manufacturing may affect stability. It was confirmed that mannitol and lactose mixed with sitagliptin phosphate monohydrate rapidly produced impurities. When sitagliptin phosphate monohydrate was mixed with mannitol, a hydrogen bond was formed between the amine group and the OH group of mannitol, and when it was mixed with lactose, an imine bond was formed. As a result, it was judged to be unstable when manufacturing tablets and was not used for this. However, when most sitagliptin phosphate monohydrate-excipient was stored at room temperature storage conditions (25 ± 2 °C/60 ± 5% relative humidity) and accelerated storage conditions (40 ± 2 °C/75 ± 5% relative humidity) for 4 weeks, the total amount of impurities was found to be 0.00%, and they were judged to be very stable in heat and humidity. Dapagliflozin phosphate hydrate initially showed a total amount of impurities of 0.09% and was confirmed to be 0.12% as a result of storage for 4 weeks under accelerated storage conditions (40 ± 2 °C/75 ± 5% relative humidity). In addition, most dapagliflozin propanediol hydrate-excipient mixtures were stable. However, the dapagliflozin phosphate hydrate-citric acid mixture showed a total amount of impurities of 0.08% at the initial stage, and a total amount of impurities of 4.34% that was higher than the standard under was found under accelerated storage conditions (40 ± 2 °C/75 ± 5% relative humidity) for 4 weeks. Finally, the sitagliptin phosphate monohydrate-dapagliflozin propanediol hydrate FDC tablet was prepared using microcrystalline cellulose, silicified microcrystalline cellulose, calcium hydrogen phosphate anhydrous, sodium starch glycolate, crospovidone, magnesium stearate, colloidal silicon dioxide, sodium stearyl fumarate, hydroxypropyl cellulose, and OPADRY II, excluding low pH excipients.

### 3.4. Risk Assessment for the Compressibility Study

**Figure 8:**
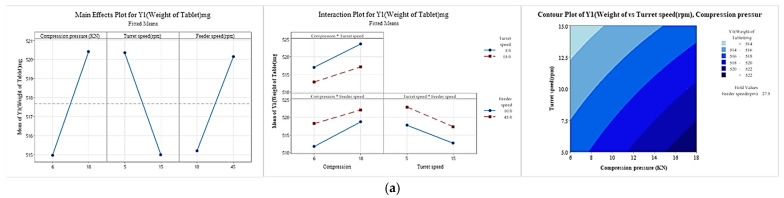

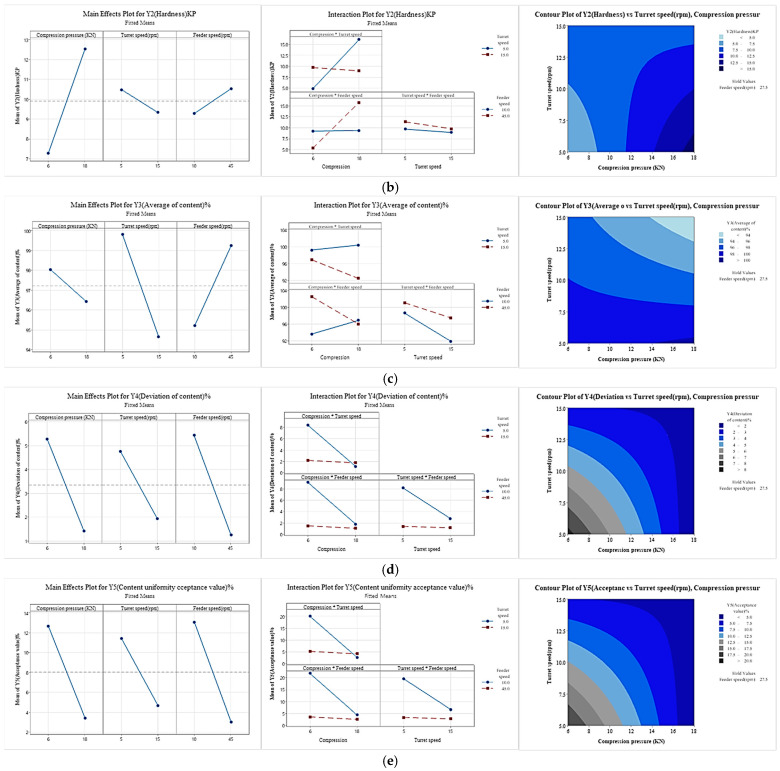
Effects of CPPs depicted by the main effect plots, interaction plots, and contour plots. (**a**) Y1 weight of tablet (mg), (**b**) Y2 hardness (KP), (**c**) Y3 average of content (%), (**d**) Y4 deviation of content (%), (**e**) Y5 content uniformity acceptance value (%).

**Figure 9:**
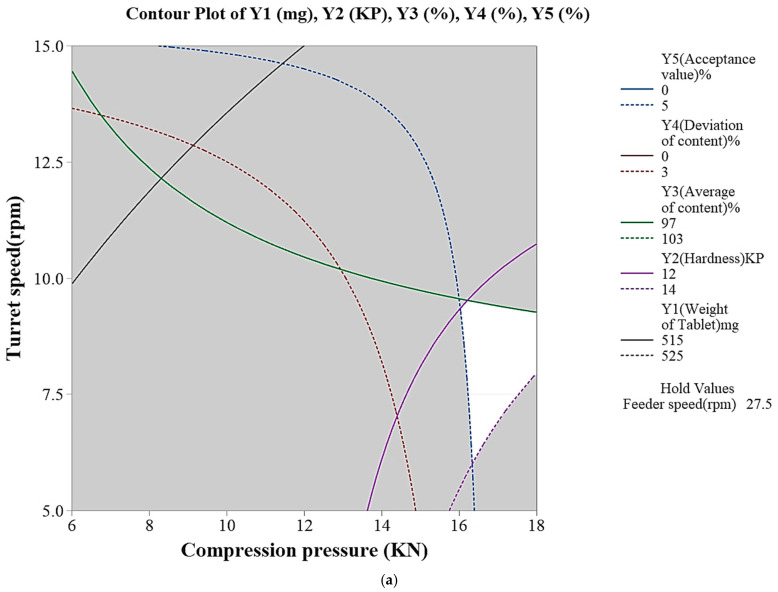

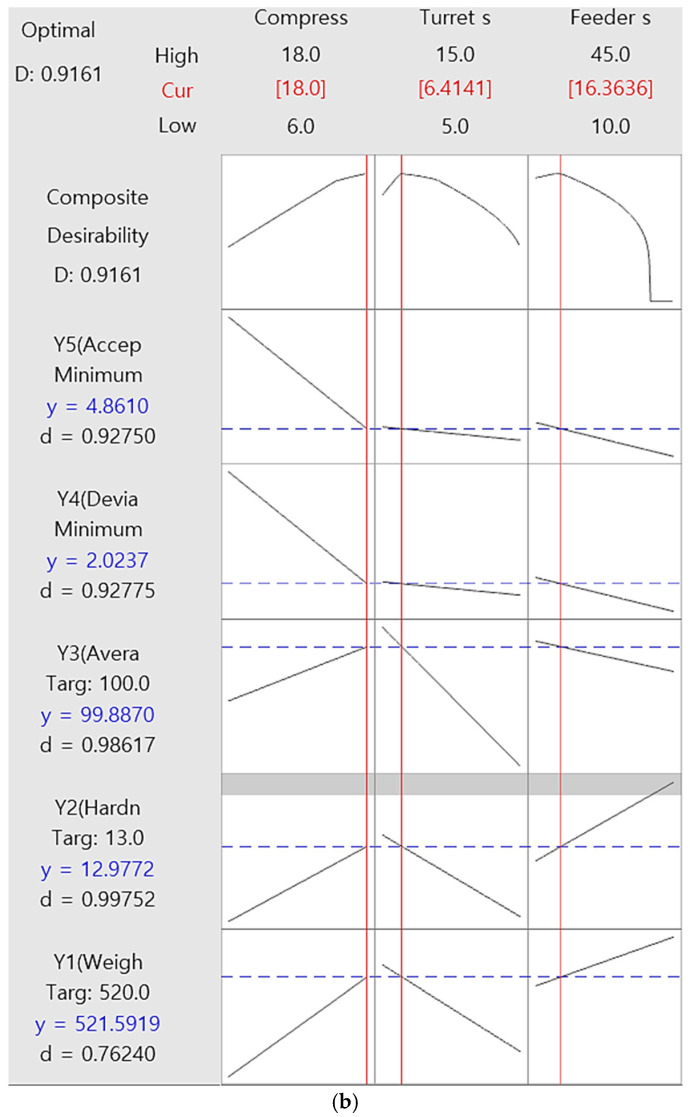
Effects of CPPs depicted by (**a**) overlaid contour plots and (**b**) response surface plots.

To manufacture Quality by Design (QbD) applicable drugs, risk assessment was applied for compressibility studies. As shown in Table 8 and Table 9, CPPs were selected by applying PHA and FMEA, which are quality risk assessment tools. Compression pressure, turret speed, and feeder speed were selected as CPPs that affect the tableting process according to the RPN score, and they were written in the order of red, green, and yellow according to the degree of risk. Based on prior research and experience, compression pressure affects hardness in the tableting process, and in particular, the turret and feeder speeds are connected to problems such as content non-uniformity, content deviation, and weight deviation; therefore, we attempted to control for these issues by setting standards. If the turret and feeder speeds are too slow or fast, content deviation and non-uniformity occur, which affects drug compliance and clinical study. Furthermore, if the compression pressure is too high, lamination and capping will occur. Therefore, the DoE was applied to set an appropriate range for the selected CPP. In this process, analysis of variance (ANOVA) and regression equations were used for the selected CPP, and response surface design analysis was conducted by optimizing significant factors. In addition, the selected effect factors were determined through standardized Pareto charts and residual plots. Through the derived results, it was confirmed that the *p*-value was 0.02 during the DoE analysis, and the *p*-value was statistically significant below 0.05. The data followed the normal distribution and the residuals appeared evenly, and the model was judged to be suitable because there was no tendency for the experimental order. After manufacturing a total of 8 formulations with optimized DoE modeling, main effect, interaction, contour, overlaid contour, and response surface plots for each of the 5 response values (Y 1 to 5) are shown in Figure 8 and Figure 9. As shown in the Main Effect plot and Interaction plot, the results were interpreted according to whether compression pressure, turret speed, and feeder speed affect the weight of tablet (mg), hardness (Kp), average of content (%), deviation of content (%), and content uniformity acceptance value (%). The weight range of the tablets was set to 515–525 mg, the hardness to 12–14 kp, the average of content to 97–103%, the deviation of content within 3%, and the content uniformity acceptance value within 5%. In addition, the DS was derived through an overlaid contour plot. The space marked in white in the DS represents the actual design space. A compression pressure of 18 KN, a turret speed of 6 rpm, and a feeder speed of 16 rpm were confirmed using response surface plots and were manufactured using the confirmed process conditions. The management of content uniformity during the tableting process was the most important factor; thus, in-process control was continuously performed. In a scale-up study, the sitagliptin phosphate monohydrate-dapagliflozin propanediol hydrate FDC double-layer tablet was manufactured by applying QbD reduced quality error and was judged to improve the drug compliance of the patients.

### 3.5. Quality Evaluation of Three Dosage Forms for Granules and Tablets

The flowability of granules and mixtures is an important factor in developing suitable equipment and process designs. Prior to tableting, flowability was evaluated by performing bulk density, tapped density, CI, and the HR. Table 10 shows the effect of flowability on the bulk density and tapped density of granules with different particle sizes. M1–M3 applied a process of dividing and mixing dapagliflozin propanediol hydrate with silicified microcrystalline cellulose several times. The CI values of the M1, M2, and M3 mixtures were 31%, 29%, and 21%, respectively; electrostatic was prevented, and flowability improved as the number of blending processes increased. Through this, as the number of blending processes increased, dapagliflozin was combined with microcrystalline cellulose to reduce the classification due to the difference in particle distribution and increase the cohesion, thereby increasing the degree of mixing. In addition, the loss of the API was reduced by preventing the occurrence of electrostatic, and uniform filling, continuous movement of the mixture, tableting, and layer separation between particles were improved. Then, single-layer, double-layer, and dry-coated tablets were prepared using a dapagliflozin propanediol hydrate mixture (M3) and sitagliptin phosphate monohydrate granules, and the results of the flowability test are shown in Table 11. The hardness, disintegration, and friability of the single-layer, double-layer, and dry-coated tablets were an average of 12 to 13 kp, within 3 min, and within 0.1% of the standard value, respectively (Table 11). In addition, the flowability of all three dosage forms was classified as ‘passable flow’. The flowability of F1–F3 was shown to be better than that of the double-layer and dry-coated tablets, but polarization occurred in the feeder during the tableting process due to the difference in particle size between the granules and the mixture. The flowability and hardness values of F7–F9 were suitable, but it was confirmed that lamination and capping of the outer layer occurred. Furthermore, as the ratio of silicified microcrystalline cellulose increased, the CI value of the dapagliflozin propanediol hydrate mixture decreased from 26% to 23%, and as the ratio of microcrystalline cellulose increased, the CI value of sitagliptin phosphate monohydrate granules decreased from 23% to 21%. As a result, it was confirmed that the flowability improved as the ratio of silicified microcrystalline cellulose and microcrystalline cellulose increased. In conclusion, among the F1–F9 FDC tablets, the F6 FDC dry-coated tablet was judged to be the optimal tablet because of its excellent flowability of granules in the feeder and tableting performance.

### 3.6. In Vitro Drug Release Pattern Study

**Figure 10:**
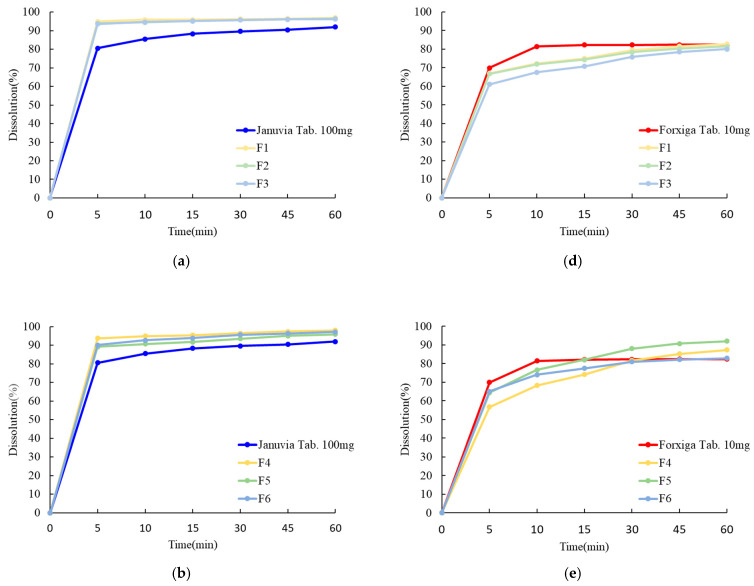

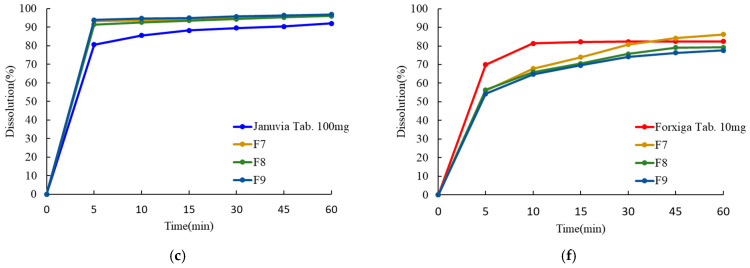
Dissolution profiles of the reference drug and sitagliptin-dapagliflozin FDC tablets in pH 6.8 medium (*n* = 6). (**a**) Single-layer tablet of sitagliptin. (**b**) Double-layer tablet of sitagliptin. (**c**) Dry-coated tablet of sitagliptin. (**d**) Single-layer tablet of dapagliflozin. (**e**) Double-layer tablet of dapagliflozin. (**f**) Dry-coated tablet of dapagliflozin.

In vitro drug release tests were conducted to compare and verify dissolution patterns and the similarity of the dissolution rates of Januvia (100 mg tab), Forxiga (10 mg tab), single-layer tablets (F1–F3), double-layer tablets (F4–F6), and dry-coated tablets (F7–F9). The dissolution profile result is shown in Figure 10. As a result of studies on single-layer, double-layer, and dry-coated tablets, the drug release rate of sitagliptin phosphate monohydrate was over 80% within 30 min. However, dapagliflozin propanediol hydrate showed a drug release rate of less than 80% within 30 min in single-layer and dry-coated tablets (Table 12). In the dry-coated tablets, sitagliptin in the outer layer was rapidly dissolved, but dapagliflozin in the core tablet was not completely disintegrated due to the accumulation of excipients in the outer layer, and it was confirmed that the dissolution pattern was remarkably low. In addition, the ratio of silicified microcrystalline cellulose and microcrystalline cellulose and the presence or absence of hydroxypropyl cellulose did not have significant effects. The dissolution profile of the three dosage forms showed that the similarity factor (f2) value according to the ICH guidelines was more than 50% (Table 13). Among them, the similarity factors of sitagliptin phosphate monohydrate and dapagliflozin propanediol hydrate of the F6 FDC double-layer tablet were 64.05% and 75.84%, respectively, which were judged to be the most similar to the reference drug. Finally, it was confirmed that the F6 FDC double-layer tablet was an optimal tablet as an immediate-release tablet showing a dissolution rate similar to that of the reference drug and a dissolution rate of 80% or more within 30 min.

### 3.7. Stability Study

Single-layer tablets, double-layer tablets, and dry-coated tablets were tested for assay and impurity for 3 months under room temperature storage conditions (25 ± 2 °C/60 ± 5% relative humidity) and accelerated storage conditions (40 ± 2 °C/75 ± 5% relative humidity); these findings are shown in Table 14 and Table 15. The assay test standards of sitagliptin and dapagliflozin were set at 90.0–110.0%. The standards for the impurity test were set at 0.5% or less of hydroxy dapagliflozin impurity, 0.2% or less of any unspecified impurity, and 2.0% or less of the total impurities.

The content of single-layer tablets, double-layer tablets, and dry-coated tablets was within 90 to 110%, and no significant differences were found. However, it was confirmed that the single-layered tablet was classified in the feeder due to the difference in particle size, resulting in content variation. In addition, since the position of the core tablet of the dry-coated tablets was not uniform, high content variation occurred. As a result of confirming the total impurities in single-layer tablets, double-layer tablets, and dry-coated tablets, the dry-coated tablets were stable for 3 months at room temperature storage conditions (25 ± 2 °C/60 ± 5% relative humidity), but the total impurities gradually increased at accelerated storage conditions (40 ± 2 °C/75 ± 5% relative humidity). In particular, single-layer tablets may have advantages in economic terms. However, due to the interaction between the APIs, the total impurities rapidly increase from 2.1% to 3.3% for 3 months under accelerated storage conditions (40 ± 2 °C/75 ± 5% relative humidity); thus, there is a concern about side effects in the body when taking the drug. However, the F6 FDC double-layer tablet had no significant occurrence of impurities, with total impurities of 0.04%, 0.05%, and 0.13%, respectively, for the initial to 3 months under room temperature storage conditions (25 ± 2 °C/60 ± 5% relative humidity) and accelerated storage conditions (40 ± 2 °C/75 ± 5% relative humidity), and it was confirmed that they were stable compared with the reference drug. These results showed improved stability of the developed sitagliptin phosphate monohydrate-dapagliflozin propanediol hydrate FDC double-layer tablet compared to the reference drug, and it was judged that there was no drug-drug interaction when stored for 24 months under room temperature storage conditions (25 ± 2 °C/60 ± 5% relative humidity).

Based on the above results, the F6 FDC double-layer tablet was selected as the optimal dosage form, and the content uniformity, assay, impurity, and dissolution tests were conducted for 9 months under room temperature storage conditions (25 ± 2 °C/60 ± 5% relative humidity) and 6 months under accelerated storage conditions (40 ± 2 °C/75 ± 5% relative humidity); the results are listed in Table 16. The content uniformity test showed within 15% of the judgment value, and the dissolution test showed a dissolution rate of 80% or more within 30 min. In addition, it was confirmed that impurities were not generated and that the stability was very high, and it was judged that high-density polyethylene (HDPE) bottles and press through pack (PTP) packaging were possible. Finally, the stability of the sitagliptin phosphate monohydrate-dapagliflozin propanediol hydrate FDC double-layer tablet was confirmed, and the drug compliance of patients may be secured by replacing coadministration of drugs with a single tablet.

### 3.8. Human Bioequivalence Study

In a phase 1 clinical trial, a single dose of sitagliptin phosphate monohydrate-dapagliflozin propanediol hydrate FDC double-layer tablet (F6) and the coadministration of the reference drugs (Forxiga 10 mg tab (dapagliflozin propanediol hydrate) and Januvia 100 mg tab (sitagliptin phosphate monohydrate)) were used, and plasma concentrations were measured up to 24 h after administration to evaluate the bioequivalence of the two groups. In addition, sitagliptin and dapagliflozin in plasma were evaluated for linearity and suitability of quality control samples. The sensitivity of the analyte showed a signal-to-noise ratio of 5 or more at the lower limit of quantification (LoQ). The concentration ranges of sitagliptin and dapagliflozin were 2–2000 ng/mL and 0.5–500 ng/mL, respectively, and the correlation coefficient value was 0.9950 or higher, showing good linearity. In addition, we evaluated the accuracy of quality control samples to confirm the suitability of analysis batches and measurements.

**Figure 11:**
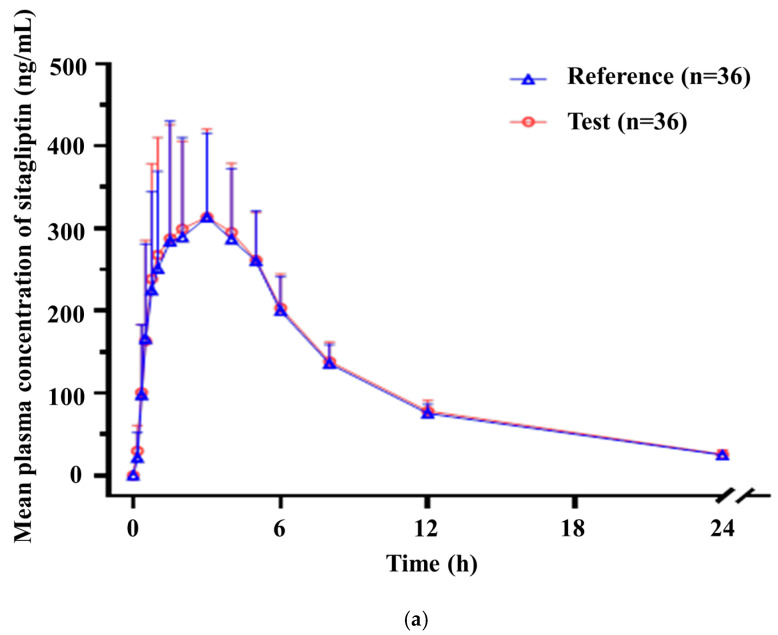

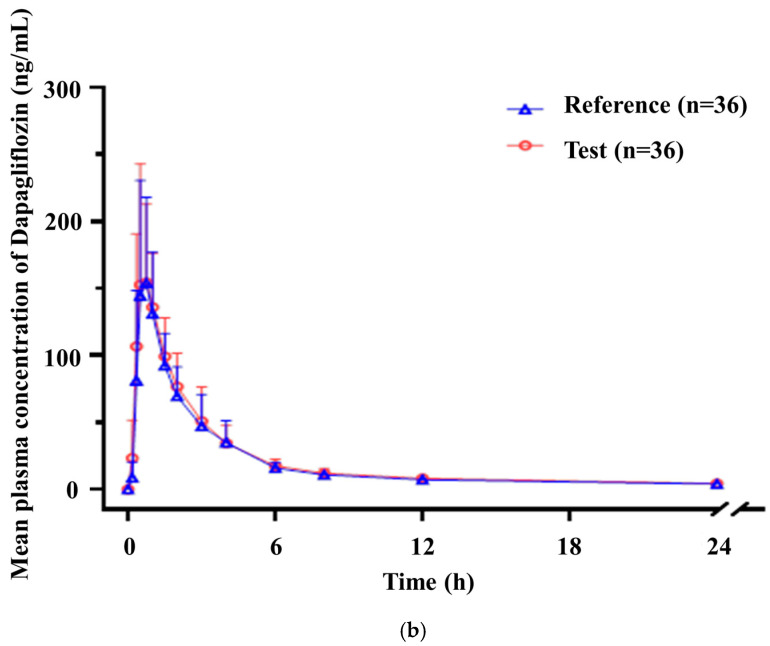
Mean plasma concentration of (**a**) sitagliptin and (**b**) dapagliflozin from 0 h to 24 h after administration (*n* = 36).

Figure 11 shows the average concentration-time graph and individual characteristics by the administration group of sitagliptin phosphate monohydrate after scheduled blood collection, and the pharmacokinetic parameters are shown in Table 17. In the case of the geometric mean of the Cmax and AUC0-t in this study, the Cmax was 412.1838 ng/mL and 421.7103 ng/mL for the reference drug and test drug, respectively, and the AUC0-t was confirmed to be 3050.0067 h*ng/mL and 3135.4766 h*ng/mL for the reference drug and test drug, respectively. When determining the 90% confidence interval of the geometric mean ratio (GMR) of the Cmax and AUC0-t to confirm whether there is a difference in exposure to the body after absorption of sitagliptin phosphate monohydrate between Januvia (100 mg tab) and the test drug, the Cmax was 1.0231 (0.9599–1.0904), and the AUC0-t was 1.0280 (1.0148–1.0414).

Figure 11 shows the average concentration-time graph and individual characteristics by the administration group of dapagliflozin propanediol hydrate after scheduled blood collection, and the pharmacokinetic parameters are shown in Table 17. In the case of the geometric mean of the Cmax and AUC0-t in this study, the Cmax was 174.0522 ng/mL and 179.7842 ng/mL for the reference drug and test drug, respectively, and the AUC0-t was confirmed to be 484.1337 h*ng/mL and 530.9329 h*ng/mL for the reference drug and test drug, respectively. When determining the 90% confidence interval of the GMR of the Cmax and AUC0-t to confirm whether there is a difference in exposure to the body after absorption of dapagliflozin propanediol hydrate between Forxiga (10 mg tab) and the test drug, the Cmax was 1.0329 (0.9305–1.1466), and the AUC0-t was 1.0967 (1.0723–1.1215).

Therefore, in this clinical trial, the Cmax and AUC0-t of sitagliptin and dapagliflozin satisfied the bioequivalence test criteria that should be within log 0.8 to log 1.25. In addition, the fact that no pharmacokinetic difference was found in the GMR for the Cmax between a single dose of the sitagliptin phosphate monohydrate-dapagliflozin propanediol hydrate FDC double-layer tablet and the reference drug indicates that it is similar to or no different in its pharmacokinetic properties in the absorption phase from the co-administered reference drug. In addition, the fact that the GMR for the AUC0-t of a single dose of sitagliptin phosphate monohydrate and dapagliflozin propanediol hydrate was equivalent indicates that it is similar or no different in its exposure to the body after absorption from the co-administered reference drug.

## 4. Conclusions

Through this study, an optimal drug release formulation with human clinical bioequivalence was derived in the development of sitagliptin phosphate monohydrate-dapagliflozin propanediol hydrate FDC tablet for the treatment of type 2 diabetes. To derive the most appropriate drug release formulation for clinical trials, we prepared single-layer tablets, double-layer tablets, and dry-coated tablets and comparatively evaluated drug release ability, manufacturability, quality, and stability. Due to the drug release pattern or stability problems caused by the interaction between the AIPs of the FDC tablet, it is important to overcome these interaction problems between the APIs and demonstrate their synergy; thus, preformulation studies and compatibility tests were conducted. Through a solubility test, it was confirmed that sitagliptin and dapagliflozin had very appropriate solubility for the immediate-release tablets to operate in 500 to 1500 mL of gastric fluid, so there was no problem in preparing immediate-release tablets. In addition, the optimized sitagliptin phosphate monohydrate-dapagliflozin propanediol hydrate FDC tablets were composed of microcrystalline cellulose, silicified microcrystalline cellulose, calcium hydrogen phosphate anhydrous, sodium starch glycolate, crospovidone, magnesium stearate, colloidal silicon dioxide, sodium stearyl fumarate, hydroxypropyl cellulose, and OPADRY, excluding low pH excipients through a compatibility test. To select suitable equipment and process design, granules and mixtures were measured for flowability, and tablets were measured for hardness, disintegration, and friability. The flowability of dapagliflozin propanediol hydrate improved as the process of dividing and mixing silicified microcrystalline cellulose several times increased. In addition, the hardness, disintegration, and friability of the single-layer tablets, double-layer tablets, and dry-coated tablets were 12 to 13 kp, within 3 min, and within 0.1%, respectively, and it was judged that they could be manufactured as FDC tablets. As a result of comparing of the dissolution pattern and the similarity factor between the three dosage forms and the reference drug through an in vitro drug release test, the dissolution rate of sitagliptin phosphate monohydrate was greater than 80% within 30 min. However, the dissolution rate of dapagliflozin propanediol hydrate in single-layer tablets and dry-coated tablets was less than 80% within 30 min. The sitagliptin phosphate monohydrate-dapagliflozin propanediol hydrate FDC double-layer tablet (F6) showed the optimal dissolution rate, and the similarity factor value according to the ICH guidelines was determined to be the most similar to that of the reference drug and was selected as the optimal formulation. In addition, through assay, dissolution, content uniformity, and impurity tests, it was confirmed that the sitagliptin phosphate monohydrate-dapagliflozin propanediol hydrate FDC double-layer tablet was stable for 9 months under room temperature storage conditions (25 ± 2 °C/60 ± 5% relative humidity) and accelerated storage conditions (40 ± 2 °C/75 ± 5% relative humidity) compared with the reference drug. In conclusion, the F6 tablet showed no drug-drug interaction when it was stored at room temperature for 24 months. Subsequent clinical trial results showed that there was no pharmacokinetic difference between the test drug and the reference drug, indicating that the pharmacokinetic properties were similar or not different in the absorption phase and exposure to the body between a single dose of the test drug and the co-administered reference drug. Therefore, this study is expected to secure safety, stability, and efficacy; simplify the number of medicines taken by patients; and reduce drug expenditures by developing an optimal dosage form through a comparative study of single-layer tablets, double-layer tablets, and dry-coated tablets.

## Figures and Tables

**Table 1:** Composition of sitagliptin phosphate monohydrate-dapagliflozin propanediol hydrate FDC tablets (*w*/*w*%).

Ingredient	Single-Layer Tablet			Double-Layer Tablet			Dry-Coated Tablet		
	F1	F2	F3	F4	F5	F6	F7	F8	F9
Sitagliptin phosphate monohydrate	38.08	44.43	38.08	38.08	44.43	38.08	38.08	44.43	38.08
Microcrystalline cellulose	43.21	38.86	45.21	43.21	38.86	45.21	43.21	38.86	45.21
Calcium hydrogen phosphate, anhydrous	10.00	10.00	10.00	10.00	10.00	10.00	10.00	10.00	10.00
Sodium starch glycolate	5.00	5.00	5.00	5.00	5.00	5.00	5.00	5.00	5.00
Hydroxypropyl cellulose	2.00	-	-	2.00	-	-	2.00	-	-
Sodium stearyl fumarate	1.71	1.71	1.71	1.71	1.71	1.71	1.71	1.71	1.71
Dapagliflozin propanediol hydrate	6.39	8.51	6.39	6.39	8.51	6.39	6.39	8.51	6.39
Silicified microcrystalline cellulose	85.12	84.99	87.12	85.12	84.99	87.12	85.12	84.99	87.12
Crospovidone	5.00	5.00	5.00	5.00	5.00	5.00	5.00	5.00	5.00
Hydroxypropyl cellulose	2.00	-	-	2.00	-	-	2.00	-	-
Colloidal silicon dioxide	0.50	0.50	0.50	0.50	0.50	0.50	0.50	0.50	0.50
Magnesium stearate	1.00	1.00	1.00	1.00	1.00	1.00	1.00	1.00	1.00

**Table 2:** Conditions of the tableting process as planned using the DoE.

Run Order	X1	X2	X3
	Compression Pressure (KN)	Turret Speed (rpm)	Feeder Speed (rpm)
1	6	15	45
2	18	5	45
3	6	5	10
4	6	15	10
5	6	5	45
6	18	5	10
7	18	15	10
8	18	15	45

**Table 3:** Solubility of sitagliptin phosphate monohydrate in various solvents.

Solvent	Apparent Solubility Test	Equilibrium Solubility Test (mg/mL)		
		Initial	12 h	24 h
Water	***	1.44	1.42	1.42
Ethanol	*	0.01	0.01	0.01
Methanol	*	0.04	0.04	0.04
Acetonitrile	*	0.00	0.00	0.00
pH 1.2	***	1.88	1.94	1.94
pH 2.0	***	3.11	3.11	3.11
pH 3.0	***	1.65	1.70	1.70
pH 4.0	***	1.54	1.61	1.61
pH 5.0	***	1.64	1.51	1.52
pH 6.0	***	1.19	1.16	1.16
pH 6.8	***	2.71	2.67	22.67
pH 7.0	***	2.82	2.76	2.76
pH 8.0	***	1.57	1.42	1.43
pH 9.0	***	1.62	1.50	1.5
pH 10.0	**	0.92	0.96	0.97
pH 11.0	***	1.56	1.44	1.44
pH 12.0	***	1.64	1.61	1.60

*: Practically insoluble, **: Very slightly soluble, ***: Slightly soluble, ****: Sparingly soluble, *****: soluble, ******: Freely soluble, *******: Very soluble.

**Table 4:** Solubility of dapagliflozin propanediol hydrate in various solvents.

Solvent	Apparent Solubility Test	Equilibrium Solubility Test (mg/mL)		
		Initial	12 h	24 h
Water	**	0.28	0.29	0.29
Ethanol	*****	75.15	78.69	78.68
Methanol	******	101.95	104.29	104.41
Acetonitrile	*****	86.03	87.39	87.41
pH 1.2	**	0.28	0.34	0.34
pH 2.0	**	0.26	0.35	0.35
pH 3.0	**	0.27	0.40	0.40
pH 4.0	**	0.30	0.37	0.37
pH 5.0	**	0.37	0.37	0.37
pH 6.0	**	0.30	0.32	0.32
pH 6.8	**	0.27	0.33	0.33
pH 7.0	**	0.30	0.32	0.32
pH 8.0	**	0.37	0.35	0.35
pH 9.0	**	0.28	0.34	0.34
pH 10.0	**	0.20	0.34	0.34
pH 11.0	**	0.14	0.21	0.21
pH 12.0	**	0.17	0.24	0.24

*: Practically insoluble, **: Very slightly soluble, ***: Slightly soluble, ****: Sparingly soluble, *****: soluble, ******: Freely soluble, *******: Very soluble.

**Table 5:** Stability studies result of impurities in pH buffer solutions for 7 days.

	Buffer	Total Impurity (%)				
		Initial	20 ± 2 °C/60 ± 5% RH		40 ± 2 °C/75 ± 5% RH	
			3 Days	7 Days	3 Days	7 Days
Sitagliptin	pH 1.2	0.02	0.01	0.09	0.02	0.14
	pH 2.0	0.02	0.03	0.23	0.08	0.59
	pH 3.0	0.08	0.08	1.42	0.37	5.64
	pH 4.0	0.02	0.00	0.01	0.00	0.02
	pH 5.0	0.03	0.00	0.03	0.00	0.03
	pH 6.0	0.05	0.00	0.03	0.01	0.06
	pH 6.8	0.04	0.01	0.06	0.00	0.03
	pH 7.0	0.00	0.00	0.03	0.00	0.03
	pH 8.0	0.06	0.01	0.03	0.00	0.03
	pH 9.0	0.00	0.00	0.00	0.00	0.03
	pH 10.0	0.02	0.00	0.03	0.00	0.03
	pH 11.0	0.04	0.00	0.03	0.00	0.04
	pH 12.0	0.03	0.00	0.03	0.00	0.03
Dapagliflozin	pH 1.2	0.06	0.08	0.12	0.11	0.73
	pH 2.0	0.00	0.00	0.00	0.02	1.03
	pH 3.0	0.06	1.30	1.99	7.29	14.76
	pH 4.0	0.00	0.00	0.01	0.04	0.12
	pH 5.0	0.00	0.16	0.26	1.36	4.48
	pH 6.0	0.00	0.00	0.04	0.15	0.28
	pH 6.8	0.00	0.01	0.01	0.00	0.12
	pH 7.0	0.00	0.02	0.04	0.7	0.69
	pH 8.0	0.00	0.03	0.05	0.42	1.06
	pH 9.0	1.02	1.27	1.47	3.90	6.31
	pH 10.0	0.15	0.46	0.66	0.30	4.78
	pH 11.0	0.00	0.14	0.22	1.99	3.69
	pH 12.0	0.00	0.75	1.05	2.62	4.11

**Table 6:** Results of compatibility testing between sitagliptin phosphate monohydrate and various excipients.

Items	Total Impurities (%)				
	Initial	20 ± 2 °C/60 ± 5% RH		40 ± 2 °C/75 ± 5% RH	
		2 Weeks	4 Weeks	2 Weeks	4 Weeks
Sitagliptin	0.00	0.00	0.00	0.00	0.00
Sitagliptin: Dapagliflozin 1:1 (*w*/*w*)	0.33	1.22	1.32	1.36	1.34
Sitagliptin: Dapagliflozin 5:1 (*w*/*w*)	0.19	0.21	0.34	0.37	0.50
Sitagliptin: Dapagliflozin 10:1 (*w*/*w*)	0.10	0.13	0.15	0.21	0.34
Sitagliptin: Mannitol 1:1 (*w*/*w*)	0.00	0.00	0.00	0.24	0.41
Sitagliptin: Microcrystalline cellulose 1:1 (*w*/*w*)	0.00	0.00	0.00	0.00	0.00
Sitagliptin: Lactose 1:1 (*w*/*w*)	0.01	0.01	0.00	0.31	0.47
Sitagliptin: Silicified microcrystalline cellulose 1:1 (*w*/*w*)	0.00	0.00	0.00	0.00	0.00
Sitagliptin: Calcium hydrogen phosphate, anhydrous 1:1 (*w*/*w*)	0.00	0.00	0.00	0.00	0.00
Sitagliptin: Pregelatinized starch 1:1 (*w*/*w*)	0.00	0.00	0.00	0.00	0.00
Sitagliptin: Sodium starch glycolate 1:1 (*w*/*w*)	0.00	0.00	0.00	0.00	0.00
Sitagliptin: Crospovidone 1:1 (*w*/*w*)	0.00	0.00	0.00	0.00	0.00
Sitagliptin: Sodium croscarmellose 1:1 (*w*/*w*)	0.00	0.00	0.00	0.00	0.00
Sitagliptin: Magnesium stearate 1:1 (*w*/*w*)	0.00	0.00	0.00	0.00	0.00
Sitagliptin: Colloidal silicon dioxide 1:1 (*w*/*w*)	0.00	0.00	0.00	0.00	0.00
Sitagliptin: Sodium stearyl fumarate 1:1 (*w*/*w*)	0.00	0.00	0.00	0.00	0.00
Sitagliptin: Hydroxypropyl cellulose 1:1 (*w*/*w*)	0.00	0.00	0.00	0.00	0.00
Sitagliptin: Calcium hydroxide 1:1 (*w*/*w*)	0.00	0.00	0.00	0.00	0.00
Sitagliptin: Citric acid 1:1 (*w*/*w*)	0.00	0.00	0.00	0.00	0.00
Sitagliptin: OPADRY II Pink 1:1 (*w*/*w*)	0.00	0.00	0.00	0.00	0.00

**Table 7:** Results of compatibility testing between dapagliflozin propanediol hydrate and various excipients.

Items	Total Impurities (%)				
	Initial	20 ± 2 °C/60 ± 5% RH		40 ± 2 °C/75 ± 5% RH	
		2 Weeks	4 Weeks	2 Weeks	4 Weeks
Dapagliflozin	0.09	0.10	0.09	0.08	0.12
Dapagliflozin: Mannitol 1:1 (*w*/*w*)	0.09	0.09	0.10	0.09	0.08
Dapagliflozin: Microcrystalline cellulose 1:1 (*w*/*w*)	0.11	0.11	0.07	0.08	0.09
Dapagliflozin: Lactose 1:1 (*w*/*w*)	0.10	0.09	0.09	0.09	0.09
Dapagliflozin: Silicified microcrystalline cellulose 1:1 (*w*/*w*)	0.08	0.09	0.09	0.09	0.10
Dapagliflozin: Calcium hydrogen phosphate, anhydrous 1:1 (*w*/*w*)	0.14	0.07	0.08	0.08	0.09
Dapagliflozin: Pregelatinized starch 1:1 (*w*/*w*)	0.08	0.10	0.15	0.08	0.09
Dapagliflozin: Sodium starch glycolate 1:1 (*w*/*w*)	0.08	0.08	0.07	0.08	0.09
Dapagliflozin: Crospovidone 1:1 (*w*/*w*)	0.10	0.08	0.09	0.09	0.09
Dapagliflozin: Sodium croscarmellose 1:1 (*w*/*w*)	0.10	0.08	0.07	0.09	0.12
Dapagliflozin: Magnesium stearate 1:1 (*w*/*w*)	0.10	0.08	0.09	0.07	0.09
Dapagliflozin: Colloidal silicon dioxide 1:1 (*w*/*w*)	0.10	0.08	0.08	0.08	0.09
Dapagliflozin: Sodium stearyl fumarate 1:1 (*w*/*w*)	0.10	0.08	0.09	0.08	0.10
Dapagliflozin: Hydroxypropyl cellulose 1:1 (*w*/*w*)	0.09	0.08	0.09	0.08	0.09
Dapagliflozin: Calcium hydroxide 1:1 (*w*/*w*)	0.09	0.05	0.05	0.05	0.09
Dapagliflozin: Citric acid 1:1 (*w*/*w*)	0.08	0.09	0.07	0.09	4.34
Dapagliflozin: OPADRY II Pink 1:1 (*w*/*w*)	0.08	0.09	0.09	0.07	0.11

**Table 8:** Preliminary hazard analysis in risk assessment.

CQA	Screening	Blending	Lubrication	Compression	Coating
Identification	Low	Low	Low	Low	Low
Assay	Low	Medium	Medium	High	Low
Uniformity	Low	Medium	Low	High	Low
Impurities	Low	Low	Low	Low	Low
Dissolution	Low	Low	Medium	Medium	Medium

Red represents an unacceptable risk, yellow is an acceptable risk, and green is a broadly acceptable risk.

**Table 9:** Failure mode effect analysis in risk assessment.

Unit Operation	CPPs	Failure Mode (Critical Event)	Justification of Failure Mode	P	S	D	RPN
Screening	Sifting	Larger than optimum mesh screen size	Non-uniform particle size distribution could cause content non-uniformity. Thus, efficacy and quality may be compromised.	3	2	2	12
Blending	Mixing rate (rpm & time)	Lower mixing speed and shorter time	Lower mixing speed could cause content non-uniformity. Thus, efficacy and quality may be compromised.	3	3	2	18
Lubrication	Mixing rate (rpm & time)	High than optimum screen size	Dissolution time may expand. Thus, efficacy may be compromised.	2	2	2	8
Compression	Speed of turret and feeder	Higher than optimum speed	Lamination, capping, and weight variation appear, affecting content, content uniformity, disintegration time, and content deviation. Therefore, efficacy may be compromised.	4	3	4	48
	Compression pressure	Higher than optimum force	The appearance and hardness of tablets may be affected. In addition, the disintegration and dissolution profiles may be affected. Thus, efficacy may be compromised.	3	3	4	36
Coating	Speed of coating pan	Higher than optimum speed	If the speed of the coating pan is rapid, this may cause damage to the tablet. Thus, bioavailability and efficacy may be compromised.	3	2	1	6

**Table 10:** Flowability of sitagliptin phosphate monohydrate granules and dapagliflozin propanediol hydrate powder.

Items		BD (g/mL)	TD (g/mL)	CI (%)	HR
>M1 *		0.3619	0.5248	31	1.45
M2 *		0.2521	0.3547	29	1.41
M3 *		0.4146	0.5248	21	1.27
Single-layer	F1	0.4291	0.5432	21	1.27
	F2	0.3863	0.5017	23	1.30
	F3	0.4110	0.5074	19	1.23
Double-layer (Sitagliptin)	F4	0.3713	0.4822	23	1.30
	F5	0.3816	0.4886	26	1.20
	F6	0.3971	0.5157	23	1.30
Double-layer (Dapagliflozin)	F4	0.3727	0.4778	22	1.28
	F5	0.4033	0.5238	23	1.30
	F6	0.4146	0.5248	21	1.27
Inner core	F7	0.3713	0.4822	23	1.30
	F8	0.3816	0.4886	26	1.28
	F9	0.3971	0.5157	23	1.30
Outer layer	F7	0.3727	0.4778	22	1.28
	F8	0.4033	0.5238	23	1.30
	F9	0.4146	0.5248	21	1.27

* M1, M2, and M3 means a manufacture of the dapagliflozin mixture follows Section 2.6.2.

**Table 11:** Quality assessment of sitagliptin phosphate monohydrate-dapagliflozin propanediol hydrate FDC tablets.

Items		Hardness (kp)	Disintegration (s)	Friability (%)
Single-layer tablet	F1	13.4	128	0.1
	F2	12.6	112	0.1
	F3	12.9	96	0.0
Double-layer tablet	F4	13.5	111	0.1
	F5	12.8	118	0.1
	F6	12.7	114	0.1
Inner-core tablet	F7	3.1	57	0.0
	F8	12.8	185	0.0
	F9	3.5	49	0.0
Dry-coated tablet	F7	13.5	166	0.1
	F8	3.3	51	0.0
	F9	12.7	159	0.1

**Table 12:** In vitro dissolution test of the reference drug and sitagliptin-dapagliflozin FDC tablets in pH 6.8 medium. The data are expressed as the mean ± SD (*n* = 6).

Items		5 Min	10 Min	15 Min	30 Min	45 Min	60 Min
Sitagliptin	Reference drug	80.6 ± 6.9	85.5 ± 6.5	88.3 ± 6.1	89.6 ± 5.4	90.4 ± 4.8	92.0 ± 3.4
	F1	94.9 ± 4.5	95.8 ± 3.7	95.8 ± 3.4	96.2 ± 3.2	96.4 ± 2.2	96.9 ± 1.4
	F2	94.0 ± 4.9	94.4 ± 3.6	95.2 ± 3.5	95.7 ± 3.5	96.1 ± 1.4	96.6 ± 0.2
	F3	93.6 ± 4.4	94.7 ± 3.4	95.1 ± 3.4	95.7 ± 3.3	96.1 ± 1.3	96.2 ± 1.4
	F4	93.7 ± 1.5	94.9 ± 1.1	95.4 ± 1.1	96.4 ± 0.9	97.4 ± 0.8	98.0 ± 0.7
	F5	89.2 ± 2.6	90.7 ± 2.3	91.7 ± 2.3	93.5 ± 1.8	95 ± 1.3	95.9 ± 1.4
	F6	90.1 ± 2.9	92.7 ± 0.3	93.8 ± 0.8	95.6 ± 0.9	96.3 ± 0.8	97.1 ± 0.4
	F7	93.3 ± 1.8	93.8 ± 0.7	94.4 ± 0.5	95.4 ± 0.2	96 ± 0.0	96.9 ± 0.5
	F8	91.4 ± 2.6	92.6 ± 2.8	93.5 ± 2.3	94.4 ± 2.0	95.3 ± 1.9	96 ± 1.6
	F9	93.9 ± 1.9	94.7 ± 1.2	95.0 ± 0.8	95.8 ± 0.5	96.4 ± 0.4	96.8 ± 0.3
Dapagliflozin	Reference drug	69.9 ± 8.3	81.4 ± 0.3	82.2 ± 0.7	82.3 ± 0.7	82.4 ± 0.6	82.4 ± 0.6
	F1	67.0 ± 0.5	72.3 ± 0.0	74.9 ± 0.4	79.4 ± 0.4	81.6 ± 0.7	82.7 ± 0.8
	F2	66.7 ± 0.6	71.9 ± 0.9	74.4 ± 0.6	78.4 ± 1.3	80.2 ± 1.4	81.4 ± 1.6
	F3	61.0 ± 0.4	67.5 ± 0.5	70.7 ± 0.8	75.8 ± 0.9	78.5 ± 1.3	80.0 ± 1.7
	F4	56.7 ± 0.7	68.3 ± 1.3	74.2 ± 2.0	81.7 ± 2.7	85.2 ± 4.0	87.3 ± 4.3
	F5	64.4 ± 1.9	76.6 ± 2.3	82.0 ± 2.2	88.0 ± 2.8	90.7 ± 3.0	92.1 ± 2.7
	F6	65.1 ± 2.0	74.0 ± 2.0	77.4 ± 1.9	81.0 ± 1.9	82.2 ± 2.2	82.9 ± 2.2
	F7	56.1 ± 4.1	67.8 ± 4.2	74.0 ± 3.5	80.8 ± 2.4	84.2 ± 3.0	86.2 ± 3.3
	F8	56.4 ± 4.6	65.9 ± 4.8	70.5 ± 1.6	75.7 ± 2.1	79.1 ± 2.7	79.2 ± 3.0
	F9	54.3 ± 4.6	64.8 ± 4.5	69.6 ± 2.9	74.2 ± 1.0	76.3 ± 1.0	77.6 ± 1.0

**Table 13:** Similarity factor (f2) evaluated of sitagliptin phosphate monohydrate-dapagliflozin propanediol hydrate FDC tablet.

Similarity Factor (f2)	F1	F2	F3	F4	F5	F6	F7	F8	F9
Sitagliptin phosphate monohydrate	58.37	59.92	60.04	57.62	69.25	64.05	61.38	64.75	59.74
Dapagliflozin propanediol hydrate	66.55	63.84	54.81	65.02	61.46	75.84	64.95	54.71	51.36

**Table 14:** Assay test results of the reference drug and sitagliptin phosphate monohydrate-dapagliflozin propanediol hydrate FDC tablets for 3 months (*n* = 3).

Items		Contents (%)				
		Initial	20 ± 2 °C/60 ± 5% RH		40 ± 2 °C/75 ± 5% RH	
			1 Month	3 Months	1 Month	3 Months
Sitagliptin	Reference drug	95.2	97.5	95.9	96.7	97.1
	F1	99.7	93.2	94.7	97.5	95.1
	F2	99.1	98.7	102.5	95.0	96.8
	F3	99.3	97.4	98.1	99.5	101.9
	F4	99.5	103.3	99.6	98.7	102.5
	F5	102.6	101.4	99.7	100.8	101.6
	F6	100.9	100.4	100.8	99.4	100.1
	F7	99.3	100.8	91.5	106.4	100.3
	F8	102.1	95.8	102.2	101.1	93.7
	F9	99.4	100.7	92.1	107.0	101.4
Dapagliflozin	Reference drug	92.8	95.7	97.1	94.3	94.4
	F1	96.2	97.3	95.7	94.4	98.3
	F2	102.5	100.1	97.2	95.7	99.6
	F3	97.5	98.4	100.8	94.3	102.7
	F4	105.9	101.8	103.3	97.2	105.4
	F5	106.7	100.0	103.8	105.6	99.2
	F6	99.5	100.9	101.0	99.7	100.6
	F7	106.1	100.5	103.2	105.9	99.4
	F8	89.8	91.3	88.9	94.7	93.3
	F9	102.7	100.7	100.0	101.4	104.3

**Table 15:** Impurity test results of the reference drug and sitagliptin phosphate monohydrate-dapagliflozin propanediol hydrate FDC tablets for 3 months (*n* = 3).

Items	Total Impurities (%)				
	Initial	20 ± 2 °C/60 ± 5% RH		40 ± 2 °C/75 ± 5% RH	
		1 Month	3 Months	1 Month	3 Months
Sitagliptin Reference drug	0.00	0.00	0.05	0.06	0.09
Dapagliflozin Reference drug	0.00	0.03	0.06	0.08	0.14
F1	0.05	0.03	0.57	0.12	2.53
F2	0.05	0.03	0.74	0.15	3.37
F3	0.05	0.05	0.49	0.34	2.12
F4	0.06	0.03	0.08	0.15	0.21
F5	0.07	0.06	0.09	0.07	0.16
F6	0.04	0.03	0.05	0.05	0.13
F7	0.08	0.03	0.09	0.15	0.27
F8	0.04	0.03	0.07	0.03	0.14
F9	0.08	0.03	0.07	0.15	0.22

**Table 16:** Stability test results of sitagliptin phosphate monohydrate-dapagliflozin propanediol hydrate FDC double-layer tablet (F6) (*n* = 10).

	Time (Month)	Sitagliptin Phosphate Monohydrate			Dapagliflozin Propanediol Hydrate			Total Impurities (%)
		Assay (%)	Dissolution (%)	Acceptance Value of Content Uniformity (%)	Assay (%)	Dissolution (%)	Acceptance Value of Content Uniformity (%)	
	Initial	100.90	98.19	4.02	99.50	96.62	7.18	0.05
20 ± 2 °C/60 ± 5% RH	3	102.10	103.09	-	100.60	97.65	-	0.07
	6	101.60	105.33	-	100.90	95.64	-	0.09
	9	101.38	102.12	-	100.45	97.19	-	0.10
	12	101.97	103.03	-	101.13	96.67	-	0.11
40 ± 2 °C/75 ± 5% RH	1	101.60	104.45	-	99.70	101.30	-	0.06
	3	101.50	103.07	-	98.20	99.11	-	0.12
	6	99.80	105.15	2.91	100.20	96.70	5.86	0.17

**Table 17:** GMR, 90% confidence interval and geometric mean of pharmacokinetic parameters (*n* = 36).

Treatment	Parameter	Geometric Mean		GMR	90% CI
		Reference	Test		
Sitagliptin	Cmax	412.1838	421.7103	1.0231	0.9599~1.0904
	AUC0-t	3050.0067	3135.4766	1.0280	1.0148~1.0414
Dapagliflozin	Cmax	174.0522	179.7842	1.0329	0.9305~1.1466
	AUC0-t	484.1337	530.9329	1.0967	1.0723~1.1215

## Data Availability

Not applicable.
